# Surface engineering strategies for PET-Based ligament scaffolds in anterior cruciate ligament reconstruction: a systematic review of mechanical and biological performances

**DOI:** 10.3389/fbioe.2026.1916072

**Published:** 2026-10-14

**Authors:** Luca Bertolino, Aurelio Picchi, Simonetta Resta, Paolo Oliva, Andrea Fidanza, Tommaso Bonanzinga

**Affiliations:** 1 Humanitas University, Milan, Italy; 2 Department of Life, Health and Environmental Sciences, University of L’Aquila, L’Aquila, Italy; 3 IRCCS Humanitas Research Hospital, Milan, Italy; 4 Mini-Invasive and Computer-Assisting Orthopaedic Surgery Unit, San Salvatore Hospital, L’Aquila, Italy; 5 Department of Biomedical Sciences, Humanitas University, Milan, Italy

**Keywords:** anterior cruciate ligament reconstruction, cell-material interaction, polyethylene terephthalate, preclinical studies, scaffold, surface functionalization

## Abstract

**Background/Objective:**

ACL reconstruction is among the most common orthopedic procedures, yet autografts and allografts carry significant limitations including donor site morbidity and immunological risk. PET-based synthetic grafts such as LARS were introduced to address these shortcomings, but their biologically inert surface raises concerns regarding cellular adhesion and host tissue integration. A new generation of functionalized PET scaffolds has emerged over the past decade, yet no systematic review has summarized this evidence since 2011. The present review aims to evaluate the biological and mechanical effects of novel functionalized PET scaffolds for ACL reconstruction.

**Methods:**

A systematic review was conducted in accordance with the PRISMA 2020 statement. PubMed, Embase, and Web of Science were searched up to 20 August 2026 for original studies investigating chemically or biologically modified polyethylene terephthalate grafts for Anterior cruciate ligament reconstruction and reporting *in vitro* outcomes, *in vivo* animal model data, and mechanical properties. Two independent reviewers performed screening, full-text assessment, and data extraction.

**Results:**

Seventeen studies met the inclusion criteria. Functionalization strategies included physical deposition, chemical surface modification, biomimetic mineral coatings, and hybrid composite fabrication. Across all studies, functionalized PET scaffolds generally demonstrated improved performance compared with unmodified PET controls. *In vitro*, enhanced cellular proliferation, upregulation of osteogenic markers, and increased angiogenic activity were observed. *In vivo*, improved graft–bone integration was demonstrated by higher bone volume fraction, reduced tunnel area, and decreased interface width. Post-implantation ultimate load to failure and stiffness generally favored functionalized PET scaffolds, though both parameters remained inferior to the native ACL in direct comparisons.

**Conclusion:**

Surface biofunctionalization appears to enhance the biological and mechanical performance of PET-based scaffolds for ACL reconstruction, suggesting that surface bioengineering may play an important role in promoting biological integration and functional performance. These findings indicate that functionalized PET scaffolds could represent potential donor-independent alternatives for ACL reconstruction and may warrant further investigation as synthetic ligament substitutes. However, a persistent mechanical gap *versus* native ligament tissue remains. These findings should be interpreted with caution due to methodological heterogeneity, predominant use of small animal models, and inconsistent outcome reporting across the included studies.

## 1 Introduction

Anterior cruciate ligament (ACL) injuries are among the most common and debilitating knee injuries in athletes and physically active individuals, often resulting in prolonged rehabilitation, delayed return to sport, and substantial socioeconomic burden (Gornitzky et al., 2016; Ramski et al., 2014; Abram et al., 2020). Arthroscopic ACL reconstruction (ACLR) remains the standard treatment for patients seeking to restore knee stability and return to high-demand activities (Kiapour and Murray, 2014; Jenkin et al., 2022). However, successful reconstruction depends not only on the restoration of mechanical stability but also on effective graft healing, including tendon-bone integration within the bone tunnels and intra-articular graft remodeling, a process commonly referred to as ligamentization (Migliorini et al., 2023; Alaseirlis et al., 2012; Clark et al., 2009; Giedraitis et al., 2014; Saccomanno et al., 2016; Boyd et al., 2024).

Graft selection plays a critical role in determining the biological and mechanical outcomes of ACLR (Yang et al., 2021; Runer et al., 2023; Widner et al., 2019). Currently, autografts and allografts represent the most widely used options in clinical practice. Autografts, including bone-patellar tendon-bone (BPTB), hamstring tendon, and quadriceps tendon grafts, remain the preferred choice because of their reliable incorporation and long-term clinical outcomes (Hughes et al., 2020; Mercurio et al., 2025). Nevertheless, these grafts are associated with several drawbacks, including donor-site morbidity, limited graft availability, prolonged rehabilitation, and postoperative pain (White et al., 2026). Similarly, allografts eliminate donor-site morbidity but present concerns related to tissue availability, slower biological incorporation, graft processing, and potential immunological or infectious complications (Migliorini et al., 2023; Alaseirlis et al., 2012; Clark et al., 2009; Giedraitis et al., 2014; Hulet et al., 2019).

To overcome these limitations, artificial ligaments have been developed with the aim of providing immediate mechanical stability, reducing rehabilitation time, and facilitating earlier return to sport. However, most first-generation synthetic ligaments were ultimately abandoned because of high rates of mechanical failure, wear debris generation, chronic synovitis, and degenerative joint changes (Pearce et al., 2020; Challa et al., 2025; Sreejal et al., 2011; Pillai et al., 2018). Among synthetic grafts, the Ligament Augmentation and Reconstruction System (LARS) still remains the most widely used and clinically investigated device (Dericks, 1995). Constructed from polyethylene terephthalate (PET) fibers, LARS is designed to reproduce selected anatomical and functional characteristics of the native anterior cruciate ligament, incorporating distinct intra-articular and bone tunnel components with a predefined fiber orientation (Viateau et al., 2013; Tiefenboeck et al., 2015).

Despite its favorable mechanical properties and encouraging short-to mid-term clinical results, PET remains an intrinsically bioinert material. The hydrophobic and chemically inert surface of PET limits protein adsorption, cellular attachment, tissue ingrowth, and graft-bone integration, potentially compromising long-term biological incorporation (Smol et al., 2022; Tulloch et al., 2019; Di Benedetto et al., 2020).

In this context alternative biomaterials have been investigated for ligament tissue engineering (Yu et al., 2023). Carbon nanotube-based scaffolds have demonstrated excellent mechanical strength and promising preclinical results in animal models, although concerns regarding inflammatory responses and long-term biocompatibility remain (Yamaguchi et al., 2023; Wang et al., 2023). Natural biomaterials such as silk fibroin have shown favorable cytocompatibility, controllable biodegradation, and the ability to support cell infiltration, collagen deposition, and neoligament formation (Bi et al., 2021; Bi et al., 2015). Similarly, biodegradable polymers such as poly(L-lactic acid) (PLLA) and poly(lactic-co-glycolic acid) (PLGA) have been explored because of their potential to provide temporary mechanical support while gradually being replaced by regenerated tissue (Sensini and Cristofolini, 2018; Capuana et al., 2022; Pan and Ding, 2012). Nevertheless, limitations related to mechanical durability, degradation kinetics, and inflammatory reactions associated with degradation by-products have hindered their widespread clinical application (Yoneyama et al., 1994; Zhang X. et al., 2016; Emonts et al., 2024).

Consequently, recent research efforts have increasingly focused on the development of functionalized PET-based scaffolds aimed at enhancing the biological performance of PET while preserving its mechanical properties. Through surface modification techniques, bioactive coatings, incorporation of growth factors, nanotechnology-based approaches, and advanced manufacturing strategies, these next-generation PET scaffolds aim to combine the superior mechanical performance of PET with enhanced biological integration and regenerative potential (Peng et al., 2025). Such approaches seek to promote cellular attachment, proliferation, extracellular matrix deposition, and tendon-bone healing while maintaining the structural stability required for ACL reconstruction. Despite the rapid growth of this field, the available evidence remains fragmented. Although previous reviews have addressed synthetic ACL grafts and the development of artificial ligaments (Reddy et al., 2021; Legnani et al., 2010; Li et al., 2022; U et al., 2023), the scope and objectives of these studies differ from those of the present review. In 2011 Mulford and Chen (2011), systematically evaluated PET grafts primarily from a clinical perspective, focusing on clinical outcomes, complications, and graft performance. Subsequent reviews have addressed the development of artificial ligaments and biofunctionalization strategies, reflecting the progressive shift from passive synthetic grafts toward biologically active scaffolds. However, the evidence regarding whether PET surface functionalization translates into improved biological integration and biomechanical performance remains heterogeneous.

More recently, Peng et al. (2025) provided a comprehensive narrative review of coating strategies for PET-based artificial ligaments, with particular emphasis on coating materials, surface-engineering and deposition techniques, and their intended biological functions. However, an important complementary question remains whether these functionalization strategies consistently result in measurable improvements in scaffold performance. Therefore, the present systematic review aimed to evaluate whether chemical or biological surface functionalization of PET-based synthetic grafts improves their biological, histological, graft–bone integration, and biomechanical performance compared with unmodified PET in preclinical models of ACL reconstruction and related *in vitro* cellular models. Specifically, we synthesized evidence on cellular and molecular responses, tissue integration, angiogenesis, osseointegration, and mechanical performance, while assessing the methodological quality and risk of bias of the available preclinical evidence. Understanding the relationship between graft functionalization and these biological and mechanical outcomes is essential for the rational design of next-generation bioactive synthetic ligament substitutes.

## 2 Materials and methods

### 2.1 Database search

This systematic review was conducted in accordance with the guidelines outlined by the Preferred Reporting Items for Systematic Reviews and Meta-Analysis (PRISMA) (Page et al., 2021).

A database search was conducted in PubMed, Embase, and Web of Science up to 20 August 2026, entering the following query (‘anterior cruciate ligament’ OR ACL) AND (‘synthetic graft’ OR ‘synthetic ligament’ OR ‘artificial ligament’ OR ‘synthetic biomaterial’ OR ‘electrospun’ OR nanocomposite* OR scaffold*) AND (‘polyethylene terephthalate’ OR ‘PET’) AND (Histologic* OR ‘in vitro’) AND (‘animal model’ OR ‘preclinical outcome’ OR ‘in vivo’).

### 2.2 Study selection process

Studies were included if they met the following criteria: (i) original articles investigating ACL reconstruction; (ii) articles related to chemically or biologically modified PET standard synthetic grafts; (iii) articles reporting *in vitro* outcomes such as cell attachment, proliferation, osteoblastic differentiation, or angiogenesis AND *in vivo* outcomes in animal models AND mechanical properties, whether performed *in vivo* or *in vitro*. This combination of biological and mechanical outcomes was intentionally required to ensure a comprehensive evaluation of scaffold performance and to identify grafts with characteristics potentially relevant to future clinical translation and application in humans. Moreover, since last systematic review was published by Mulford and Chen (2011) in 2011 and summarized studies published up to June 2010, only original research articles published from 2010 onward were considered eligible for this review. The temporal restriction was adopted to capture the evolution of PET biofunctionalization following the previous systematic review, while the eligibility criteria of the present review were defined independently based on the specific research question. To further ensure that relevant earlier evidence was not overlooked, an additional targeted search of the pre-2010 literature was performed using the same conceptual combination of ACL reconstruction, PET/synthetic grafts, surface modification/biofunctionalization, and biological/mechanical outcomes; this search did not identify any studies meeting all the present eligibility criteria.

Studies were excluded if they (i) were not written in English; (ii) were not original research articles, thus reviews, conference proceedings, abstracts, book chapters, or letters to the editor; (iii) investigated the reconstruction of ligaments other than the anterior cruciate ligament; (iv) investigated bioconductive scaffold or (v) involved synthetic grafts other than modified PET (e.g., polyethylene, polytetrafluoroethylene, or polyester).

The database search outputs were imported into Rayyan (Cambridge, MA, United States) for duplicate removal and screening. After the removal of duplicates, titles and abstracts were screened by two independent reviewers (LB and AP) who excluded papers that did not match eligibility criteria and retrieved the full version of the remaining ones. The full articles then underwent independent review by the same two authors, who discussed the papers and resolved any disagreements by consensus. If consensus was not reached at first, a senior author (SR) was appealed to make the final decision. Reference lists of selected articles were also checked to identify titles that matched our search criteria missed during the original search. Moreover, in case of multiple papers authored by the same study groups, reported datasets were carefully compared among the papers to avoid reporting duplicated data in the present review.

### 2.3 Data extraction and synthesis

Two reviewers (LB and AP) examined the full text of the selected articles to collect the following pieces of information and recorded them on a Microsoft Excel Spreadsheet (Microsoft Corporation, Redmond, WA, United States): first author and year, description of the PET scaffold studied, manufacturing technique, animal model, *in vitro* outcomes, *in vivo* outcomes and mechanical testing outcomes. Quantitative numerical data were extracted wherever available. However, when such data were not explicitly reported in the text but were presented graphically, the corresponding values reported in the tables without an associated ± error were extracted directly from the graphs using WebPlotDigitizer (Copyright 2010–2024, Ankit Rohatgi).

### 2.4 Risk of bias assessment

To assess the methodological quality of the included studies, the SYRCLE’s risk of bias tool for animal studies was employed (Hooijmans et al., 2014). Any discrepancy in the independent assessment was discussed and resolved by consensus. If consensus was not reached at first between the two reviewers (LB and AP), a senior author (SR) was appealed to make the final decision.

As there is no validated or consistently utilized risk of bias assessment tool for *in vitro* studies, bias could not be assessed in these studies.

## 3 Results

### 3.1 Database search and study selection

The database search resulted in 83 records, of which 36 were duplicates and were removed. The titles and abstracts of the remaining 47 articles were screened, leading to the full-text inspection of 27 articles, of which 17 were ultimately included in the present review (Figure 1).

**FIGURE 1:**
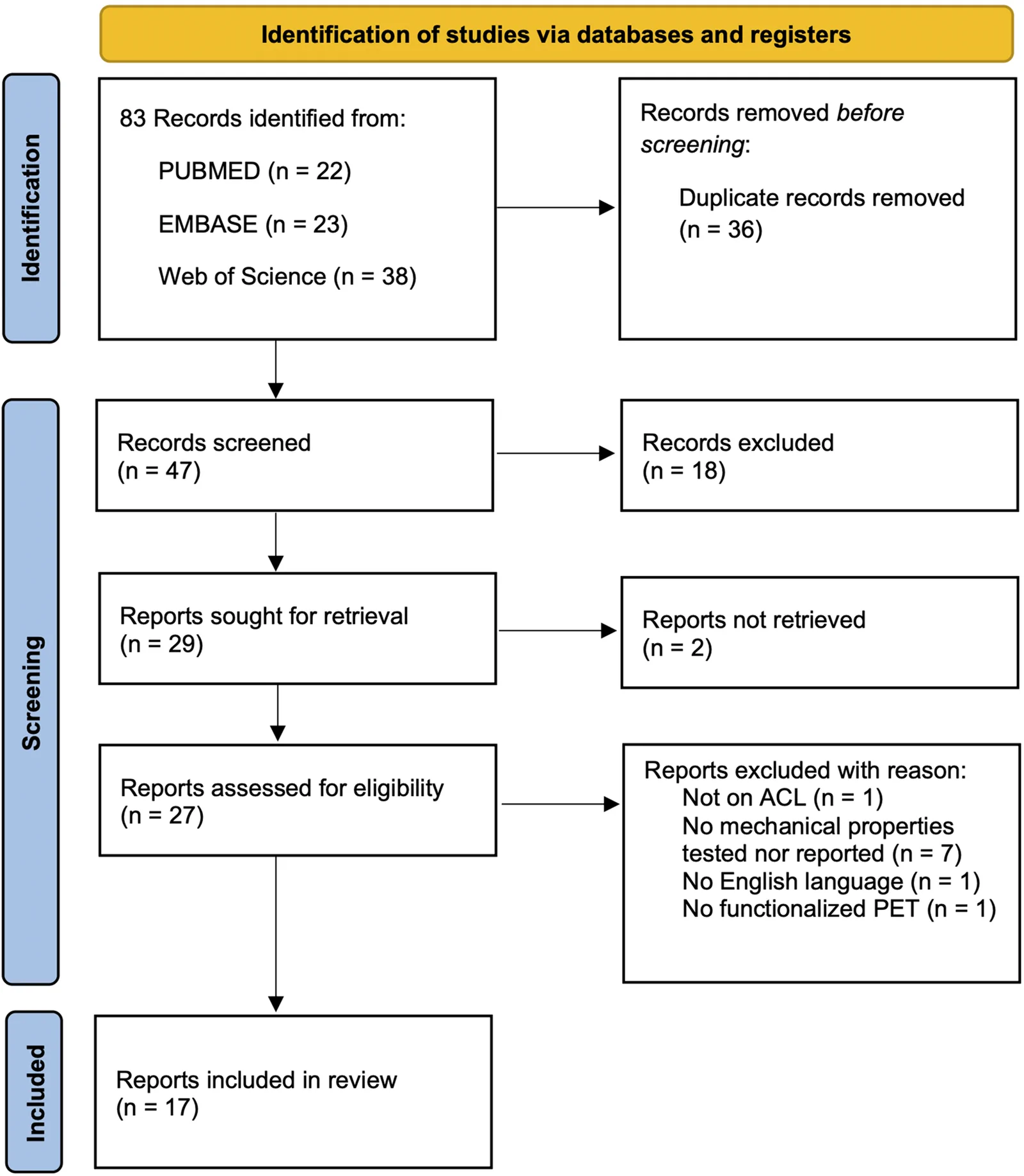
PRISMA 2020 flow diagram for new systematic reviews which included searches of databases and registers only.

### 3.2 Risk of bias assessment

Study quality assessed with SYRCLE’s risk of bias tool for animal studies is presented in Table 1.

**TABLE 1:** SYRCLE’s risk of bias tool for animal studies.

Author, year	D1	D2	D3	D4	D5	D6	D7	D8	D9	D10
(Li et al., 2012)	UNCERTAIN	LOW	UNCERTAIN	UNCERTAIN	HIGH	UNCERTAIN	UNCERTAIN	LOW	UNCERTAIN	UNCERTAIN
(Vaquette et al., 2013)	UNCERTAIN	LOW	UNCERTAIN	UNCERTAIN	HIGH	UNCERTAIN	UNCERTAIN	LOW	UNCERTAIN	UNCERTAIN
(Jiang et al., 2014)	UNCERTAIN	LOW	UNCERTAIN	UNCERTAIN	HIGH	UNCERTAIN	UNCERTAIN	LOW	UNCERTAIN	UNCERTAIN
(Li et al., 2014)	HIGH	LOW	HIGH	UNCERTAIN	HIGH	UNCERTAIN	UNCERTAIN	LOW	UNCERTAIN	UNCERTAIN
(Wang et al., 2015)	UNCERTAIN	LOW	UNCERTAIN	UNCERTAIN	HIGH	UNCERTAIN	UNCERTAIN	LOW	UNCERTAIN	UNCERTAIN
(Zhang et al., 2016)	UNCERTAIN	LOW	UNCERTAIN	UNCERTAIN	HIGH	UNCERTAIN	UNCERTAIN	LOW	UNCERTAIN	UNCERTAIN
(Ai et al., 2017)	UNCERTAIN	LOW	UNCERTAIN	UNCERTAIN	HIGH	UNCERTAIN	UNCERTAIN	LOW	UNCERTAIN	UNCERTAIN
(Li et al., 2017)	UNCERTAIN	LOW	UNCERTAIN	UNCERTAIN	HIGH	UNCERTAIN	UNCERTAIN	LOW	UNCERTAIN	UNCERTAIN
(Cai et al., 2018)	HIGH	LOW	HIGH	UNCERTAIN	HIGH	UNCERTAIN	UNCERTAIN	LOW	UNCERTAIN	UNCERTAIN
(Wang et al., 2018)	UNCERTAIN	LOW	UNCERTAIN	UNCERTAIN	HIGH	UNCERTAIN	UNCERTAIN	LOW	UNCERTAIN	UNCERTAIN
(Yang et al., 2019)	UNCERTAIN	LOW	UNCERTAIN	UNCERTAIN	HIGH	UNCERTAIN	UNCERTAIN	LOW	UNCERTAIN	UNCERTAIN
(Cai et al., 2020)	LOW	UNCERTAIN	UNCERTAIN	UNCERTAIN	HIGH	UNCERTAIN	UNCERTAIN	LOW	UNCERTAIN	UNCERTAIN
(Zhang et al., 2020)	UNCERTAIN	LOW	UNCERTAIN	UNCERTAIN	HIGH	UNCERTAIN	UNCERTAIN	LOW	UNCERTAIN	UNCERTAIN
(Cai et al., 2021)	UNCERTAIN	LOW	UNCERTAIN	UNCERTAIN	HIGH	UNCERTAIN	UNCERTAIN	LOW	UNCERTAIN	UNCERTAIN
(Kang et al., 2021)	UNCERTAIN	LOW	UNCERTAIN	UNCERTAIN	HIGH	UNCERTAIN	UNCERTAIN	LOW	UNCERTAIN	UNCERTAIN
(Ma et al., 2021)	UNCERTAIN	UNCERTAIN	UNCERTAIN	UNCERTAIN	HIGH	UNCERTAIN	UNCERTAIN	LOW	UNCERTAIN	UNCERTAIN
(Wei et al., 2026)	LOW	LOW	UNCERTAIN	UNCERTAIN	HIGH	UNCERTAIN	UNCERTAIN	LOW	UNCERTAIN	UNCERTAIN

D1, Sequence generation (random allocation of animals); D2, Baseline characteristics; D3, Allocation concealment; D4, Random housing; D5, Blinding of caregivers/investigators; D6, Random outcome assessment; D7, Blinding of outcome assessor; D8, incomplete outcome data; D9, Selective outcome reporting; D10, other source bias.

All included studies were judged to be at low risk of bias for incomplete outcome data (Li et al., 2012; Vaquette et al., 2013; Jiang et al., 2014; Li et al., 2014; Wang et al., 2015; Zhang P. et al., 2016; Ai et al., 2017; Li et al., 2017; Cai et al., 2018; Wang S. et al., 2018; Yang et al., 2019; Cai et al., 2020; Zhang et al., 2020; Cai et al., 2021; Kang et al., 2021; Ma et al., 2021; Wei et al., 2026).

In contrast, all studies were rated as having an unclear risk of bias for random housing, random outcome assessment, blinding of outcome assessors, selective outcome reporting, and other potential sources of bias, primarily due to insufficient methodological information reporting (Li et al., 2012; Vaquette et al., 2013; Jiang et al., 2014; Li et al., 2014; Wang et al., 2015; Zhang P. et al., 2016; Ai et al., 2017; Li et al., 2017; Cai et al., 2018; Wang S. et al., 2018; Yang et al., 2019; Cai et al., 2020; Zhang et al., 2020; Cai et al., 2021; Kang et al., 2021; Ma et al., 2021; Wei et al., 2026).

For sequence generation (random allocation of animals), two studies were considered at low risk of bias (Cai et al., 2020; Wei et al., 2026), and two studies were judged to be at high risk (Li et al., 2014; Cai et al., 2018). The remaining studies did not provide sufficient details on the randomization process and were therefore classified as having an unclear risk of bias (Li et al., 2012; Vaquette et al., 2013; Jiang et al., 2014; Wang et al., 2015; Zhang P. et al., 2016; Ai et al., 2017; Li et al., 2017; Wang S. et al., 2018; Yang et al., 2019; Zhang et al., 2020; Cai et al., 2021; Kang et al., 2021; Ma et al., 2021).

Regarding baseline characteristics, all but two studies were considered to be at low risk of bias, reflecting adequate comparability between experimental groups at study initiation (Li et al., 2012; Vaquette et al., 2013; Jiang et al., 2014; Li et al., 2014; Wang et al., 2015; Zhang P. et al., 2016; Ai et al., 2017; Li et al., 2017; Cai et al., 2018; Wang S. et al., 2018; Yang et al., 2019; Zhang et al., 2020; Cai et al., 2021; Kang et al., 2021; Wei et al., 2026). Two studies were rated as having an unclear risk of bias because insufficient information was provided to assess baseline group equivalence (Cai et al., 2020; Ma et al., 2021).

Allocation concealment emerged as a potential source of bias. Two studies were judged to be at high risk of bias (Li et al., 2014; Cai et al., 2018), while the remaining studies lacked sufficient information to allow a definitive assessment and were therefore categorized as having an unclear risk of bias (Li et al., 2012; Vaquette et al., 2013; Jiang et al., 2014; Wang et al., 2015; Zhang P. et al., 2016; Ai et al., 2017; Li et al., 2017; Wang S. et al., 2018; Yang et al., 2019; Cai et al., 2020; Zhang et al., 2020; Cai et al., 2021; Kang et al., 2021; Ma et al., 2021; Wei et al., 2026).

Finally, all studies were considered to be at high risk of bias regarding the blinding of caregivers and investigators, as none reported measures to ensure blinding during the conduct of the experiments (Li et al., 2012; Vaquette et al., 2013; Jiang et al., 2014; Li et al., 2014; Wang et al., 2015; Zhang P. et al., 2016; Ai et al., 2017; Li et al., 2017; Cai et al., 2018; Wang S. et al., 2018; Yang et al., 2019; Cai et al., 2020; Zhang et al., 2020; Cai et al., 2021; Kang et al., 2021; Ma et al., 2021; Wei et al., 2026).

### 3.3 Data extraction

#### 3.3.1 PET ligaments scaffold and functionalization

An overview of PET ligaments scaffold and functionalization is presented in Table 2.

**TABLE 2:** PET ligaments scaffold, functionalization, physiochemical properties and manufacturing technique overview.

Author, year	PET modification/tissue engineering strategy/scaffold type	Physicochemical properties	Manufacturing technique
(Li et al., 2012)	PET artificial ligament (LARS™) surface-modified by LBL self-assembly of HA and CA	Morphological characterization: ↑ Roughness in modified PET (SEM analysis) Wettability: ↓ water contact angle compared vs. PET	Low-temperature plasma activation followed by LBL electrostatic self-assembly of HA-CG multilayers
(Vaquette et al., 2013)	PET artificial ligament (LARS™) with PolyNaSS surface grafting	Morphological characterization: homogeneous distribution of PolyNaSS in GL (Toluidine blu staining)	Poly(NaSS) grafted onto ozonated PET surfaces *via* thermally initiated “grafting-from” radical polymerization of sodium styrene sulfonate.
(Jiang et al., 2014)	PET artificial ligament (LARS™) with Silk Fibroin coating and HAP deposition	Morphological characterization: ↓crystallinity of SF/PET vs. pure HAP (XRD analysis)	SF coating *via* immersion; HAP deposited onto the SF/PET scaffold *via* soaking process.
(Li et al., 2014)	PET artificial ligament (LARS™) with functional AKT nanocoating.	Morphological characterization: fiber-like microstructure of AKT-PET. Uniform AKT layer with nano-sized particles (higher magnification images) (SEM analysis) Amorphous phase structure for the prepared AKT-PET (XRD analysis)	Nanocoating *via* PLD
(Wang et al., 2015)	PET artificial ligaments with graphene surface-coating	Morphological characterization: No difference in porosity in G-PET-AL vs. PET-AL (liquid displacement method) Graphene wrinkles on G-PET-AL surface (SEM analysis) Wettability: ↓water contact angle G-PET-AL (53.8 ± 2.5) vs. PET-AL (83.1 ± 5.9)	Chemical vapor PMMA-assisted graphene transfer
(Zhang et al., 2016b)	PET artificial ligament (LARS™) surface-coated with collagen incorporating sustained-release simvastatin microspheres	Morphological characterization: ↑ Roughness in SIM/COL/PET vs. PET. ↑ Number of microsphere in SIM/COL/PET group (SEM analysis) Wettability: ↓ water contact angle of SIM/COL/PET (34.11 ± 2.12) vs. COL/PET (33.68 ± 1.94) and PET (85.25 ± 5.19)	Low-temperature plasma activation followed by collagen coating with simvastatin-loaded PCL microspheres
(Ai et al., 2017)	PET artificial ligament coated with silk fibroin stabilized *via* EDC/NHS-mediated crosslinking	Morphological characterization: ↑Roughness SF-EDC/PET vs. PET (SEM analysis) Wettability: ↓water contact angle in SF-EDC/PET (58.5°) vs. PET (106°)	The PET scaffolds were functionalized with silk fibroin *via* EDC/NHS-mediated crosslinking
(Li et al., 2017)	PET artificial ligament (LARS™) nanocoated with copper containing bioactive glass (Cu-BG)	Morphological characterization: uniform bioactive glass nanocoating composed of nanosized particles in Cu-BG/PET (SEM analysis) Wettability: ↓Water contact angle of Cu-BG/PET (30°) vs. PET (142°)	Nanocoating *via* PLD
(Cai et al., 2018)	PET artificial ligament with segmented coating of silk fibroin on intra-articular part (IAP) and HAP on intra-osseous part (IOP)	Morphological characterization: ↑ Roughness SF-HAP/PET vs. PET. ↑ Number of crystal particles in SF-HAP/PET (SEM analysis) Wettability: ↓ Water contact angle of PET–SF (34.73° ± 2.31°) and PET–HAP (45.90° ± 2.82°) vs. PET (114.10° ± 0.92°).	Segmented surface modification. SF coating on IAP *via* crosslinking with EDC/NHS; HAP coating on IOP *via* gelatin-mediated biomineralization (Ca–P deposition).
(Wang et al., 2018)	PET artificial ligament plasma spray-coated with HAP	Morphological characterization: ↑ Roughness of PET/HAP-plasma and PET/HAP-immersed vs. PET. Flake sHAed HAP nanoparticles in PET/HAP immersed vs. sphere sHAed nanoparticles in PET/HA-plasma. ↓ Number of the nanoparticles in the PET/HAP-immersed vs. PET/HAP-plasma. Narrow diameter distribution width of HAP nanoparticles in PET/HAP-plasma (SEM analysis) Wettability: ↓ water contact angle of PET/HAP-plasma (75.4° ± 10.4°) vs. PET/HAP-immersed (87.6° ± 10.0°) vs. PET (105.3° ± 10.9°)	HAP coating *via* thermal spraying technique
(Yang et al., 2019)	PET artificial ligament surface-modified with HPC coating to enhance graft-to-bone healing and ligamentization	Morphological characterization: Thin HPC layer on fiber surface of HPC/PET. (SEM analysis)	Corona discharge surface activation followed by high-pressure spray coating of aqueous HPC solution onto PET ligament
(Cai et al., 2020)	Biomineralizaion of PET ligaments with hydroxyapatite (HAP)	Morphological characterization: ↑ Number of crystal particles in HAP–PET fibers vs. PET (SEM). Typical PET diffraction peaks in both groups and a typical peak corresponding to pure HAP in HAP-PET. (XRD analysis) Wettability: ↓ Water contact angle of HAP–PET (45.48° ± 1.66°) vs. PET (115.20° ± 1.32°)	PET ligaments were surface-treated with NaOH and gelatin, followed by biomineralization with HAP
(Zhang et al., 2020)	PET mesh + electrospun PCL membrane + BMP-7 (hybrid “Swiss roll”)Hybrid artificial ligament composed of non-degradable PET mesh fabric combined with degradable electrospun PCL nanofibrous membrane loaded with BMP-7 in a “Swiss roll”-like structure	Morphological characterization: Layers of alternately superimposed PET fibers and electrospun nanofiber membranes in the cross-sections of PCL/PET and BMP-7/PCL/PET. Highly uniform BMP-7/PCL/PET scaffolds with randomly interconnected porous and beaded structure PAH and PSS granular complexes on PCL fibers surface (SEM Analysis). Wettability: ↑ water contact angle (by increasing the number of layers) from 90° to 108.2° but lower than PCL (112.8° ± 2.2).	The hybrid ligament was fabricated by combining electrospinning and LBL self-assembly to produce a BMP-7–loaded PCL nanofibrous membrane, which was then rolled together with a PET mesh fabric into a ‘Swiss roll’-like multilayered structure.
(Cai et al., 2021)	PET + CaP electrodeposited (ED-CaP) (vs. BM-CaP)PET artificial ligament surface-modified with CaP coating *via* electrodeposition to enhance osteogenic activity and graft–bone integration	Morphological characterization: ↑Roughness of PET/BM-CaP and PET/ED-CaP groups (with many crystal particles) vs. PET. More uniform particles of PET/ED-CaP group than PET/BM-CaP group. (SEM analysis)	Electrodeposition of calcium phosphate, ammonia-pretreated PET scaffolds using a two-electrode electrochemical system at 3 V for 2 h
(Kang et al., 2021)	PET + PDA coating + BMP-2 immobilized (500B-PDA-PET) PET artificial ligament surface-modified with PDA coating and immobilized BMP-2 to enhance bioactivity and graft-to-bone healing	Morphological characterization: Layer with fine coarse particles in PDA-PET, 250B-PDA-PET and 500B-PDA-PET modified by BMP-2 (thicker than the PDA layer in some areas). (SEM analysis) Wettability: In PDA coating or BMP-2-immobilized groups, the water drop penetrated into the surface so fast that a static water contact angle cannot be measured (better wettability compared to the PET group).	Immersion of PET scaffold in dopamine solution to form PDA coating followed by covalent immobilization of BMP-2 onto the PDA layer
(Ma et al., 2021)	PET + Sr-substituted HAP coating (2SrHAP-PET). PET artificial ligament surface-modified with strontium-substituted hydroxyapatite nanoparticle coating to enhance osteogenic bioactivity and graft–bone osseointegration	Morphological characterization: Diffraction peaks of HAP in SrHAP with different strontium contents. ↓ Crystallinity of SrHAP vs. HAP (X-ray analysis). No difference in morphology and size among SrHAP and HAP nanoparticles. Rod and dot sHPAe of HAP and SrHAP nanoparticles with good dispersion without agglomeration. (TEM analysis) Wettability: ↓water contact angle of HAP-PET (69.21° ± 2.01°) and 2SrHAP-PET (67.61° ± 1.71°) vs. PET (115.41° ± 0.91°)	Microwave-hydrothermal synthesis of SrHAP nanoparticles followed by alkaline hydrolysis pretreatment of PET and coating *via* gelatin-assisted soaking method
(Wei et al., 2026)	PET artificial ligament coated with Black Phosphorous and Silk Fibroin	Morphological characterization: ↑ Roughness in BP/SF-PET Film layer of BP/SF coating of 4–5 μm in BP/PET (SEM analysis) Typical PET diffraction peaks in both groups and a typical peak corresponding to pure BP in BP/SF-PET. (XRD analysis)	BP/SF composite coating deposited onto the alkali-treated PET fabric *via* immersion (dip-coating) process following liquid-phase exfoliation of BP and regeneration of silk fibroin.

ACL, anterior cruciate ligament; AKT, akermanite; BM-CaP, biomineralized calcium phosphate; BMP-2, Bone Morphogenetic Protein-2; BMP-7, Bone Morphogenetic Protein-7; BP, black phosphorous; BS/BV, Bone surface/bone volume; BV/TV, Bone volume/total volume; CaP, calcium phosphate; CG, cationized gelatin; EDC, 1-Ethyl-3-(3-dimethylaminopropyl) carbodiimide; ED-CaP, electrodeposited calcium phosphate; EDS, Energy-Dispersive Spectroscopy; GL, grafted ligament; HA, hyaluronic acid; HA-CG, Hyaluronic Acid-Cationized Gelatin; HAP, hydroxyapatite; HIF-1a, Hypoxia-Inducible Factor-1, alpha; HPC, hydroxypropylcellulose; IAP, intra-articular part; IOD, integrated optical density; IOP, intra-osseous part; LbL, Layer-by-Layer; NHS, N-Hydroxysuccinimide; NGL, non-grafted ligament; OCN, osteocalcin; PAH, Poly (allylamine hydrochloride); PCL, polycaprolactone; PDA, polydopamine; PET, polyethylene terephthalate; PLD, pulse laser deposition; PMMA, polymethyl methacrylate; PolyNaSS, poly (sodium styrene sulfonate); PSS, Poly(sodium 4-styrenesulfonate); SF, silk fibroin; SEM, scanning electron microscopy; SIM, simvastatin; SrHAP, Strontium-substituted hydroxyapatite; TbN, trabecular number; TbSp, trabecular spacing; TbTh, trabecular thickness; TEM, transmission electron microscopy; TGA, thermogravimetric analysis; VEGF, vascular endothelial growth factor; XPS, X-ray Photoelectron Spectroscopy; XRD, X-ray Diffraction; ↓ lower (non significant); ↓↓ significantly lower; ↑ higher (non significant); ↑↑ significantly higher.

All studies performed a morphological characterization of PET-modified scaffolds. Fifteen studies adopted electron microscopy techniques, including SEM (Li et al., 2012; Li et al., 2014; Wang et al., 2015; Zhang P. et al., 2016; Ai et al., 2017; Li et al., 2017; Cai et al., 2018; Wang S. et al., 2018; Yang et al., 2019; Cai et al., 2020; Zhang et al., 2020; Cai et al., 2021; Kang et al., 2021; Wei et al., 2026) and TEM (Ma et al., 2021). Additional techniques included XRD analysis (Jiang et al., 2014; Li et al., 2014; Cai et al., 2020; Ma et al., 2021; Wei et al., 2026), liquid displacement method to evaluate porosity (Wang et al., 2015), and toluidine staining to assess coating distribution (Vaquette et al., 2013).

SEM analysis showed that surface functionalization modified the native smooth morphology of PET in most studies. Uniform glass nanocoating was reported in one study (Li et al., 2017), while increased surface roughness was observed in six studies (Li et al., 2012; Zhang P. et al., 2016; Ai et al., 2017; Cai et al., 2018; Wang S. et al., 2018; Cai et al., 2020; Cai et al., 2021). Three studies quantified particle number (Zhang P. et al., 2016; Cai et al., 2018; Wang S. et al., 2018), and three further characterized particle shape and/or size (Wang S. et al., 2018; Cai et al., 2021; Ma et al., 2021). In particular, Wang S. et al. (2018) reported flake-shaped HAP nanoparticles in PET/HAP-immersed scaffolds and spherical nanoparticles in PET/HAP-plasma scaffolds, while Cai et al. (2021) described a more uniform particle distribution in PET/ED-CaP compared with PET/BM-CaP. TEM analysis by Ma et al. (2021) identified rod- and dot-shaped HAP and SrHAP nanoparticles, with no significant differences in morphology between the two.

Layer morphology was described in five studies (Li et al., 2014; Wang et al., 2015; Yang et al., 2019; Zhang et al., 2020; Kang et al., 2021). Yang et al. (2019) reported thin HPC layers on the fiber surface of HPC/PET, with the underlying PET maintaining smooth and homogeneous appearance. Zhang et al. (2020) described a multilayered architecture of superimposed PET fibers and electrospun nanofiber membranes in PCL/PET and BMP-7/PCL/PET scaffolds, which also showed a highly uniform, randomly interconnected porous and beaded structure. Li et al. (2014) reported a fiber-like microstructure in AKT-PET, characterized by a uniform AKT layer with nano-sized particles, while Wang et al. (2015) observed graphene-induced wrinkles on the G-PET-AL surface. Kang et al. (2021) described layered coatings with fine and coarse particles in PDA-PET, 250B-PDA-PET, and 500B-PDA-PET modified with BMP-2, with localized regions showing increased thickness compared with the PDA layer.

XRD analysis revealed that functionalization affected the crystallinity of PET-based scaffolds. Jiang et al. (2014) and Ma et al. (2021) reported reduced crystallinity in SF/PET and SrHAP compared with pure HAP, with overlapping diffraction peaks between SrHAP and HAP. Li et al. (2014) described an amorphous phase structure in AKT/PET, while Wei et al. (2026) identified both characteristic PET diffraction peaks and BP-related peaks in BP/SF-PET. Similarly, Cai et al. (2020) identified both characteristic HAP and PET diffraction peaks in HAP-PET.

Porosity evaluation was performed using the liquid displacement method by Wang et al. (2015) and showed no significant differences between G-PET-AL and PET-AL groups.

Toluidine staining by Vaquette et al. (2013) demonstrated a homogeneous distribution of PolyNass within the grafted ligament.

Wettability, a key parameter influencing scaffold biocompatibility and bioactivity, was evaluated in 11 studies (Li et al., 2012; Wang et al., 2015; Zhang P. et al., 2016; Ai et al., 2017; Li et al., 2017; Cai et al., 2018; Wang S. et al., 2018; Zhang et al., 2020; Cai et al., 2021; Kang et al., 2021; Ma et al., 2021). In ten studies (Wang et al., 2015; Zhang P. et al., 2016; Ai et al., 2017; Li et al., 2017; Cai et al., 2018; Wang S. et al., 2018; Cai et al., 2020; Cai et al., 2021; Kang et al., 2021; Ma et al., 2021), water contact angle (WCA) was quantitatively measured, consistently showing lower values compared with pristine PET (115°), reflecting the increased hydrophilicity induced by surface functionalization. Only one study (Zhang et al., 2020) reported an increase in WCA with increasing number of layers, although values remained lower than those of pure PET or PCL (112.8° ± 2.2°). Two studies (Li et al., 2012; Kang et al., 2021) provided only qualitative assessments both indicating improved wettability compared with PET.

#### 3.3.2 Manufacturing and functionalization technique

An overview of the manufacturing methods employed across the included studies is presented in Table 2. The main functionalization strategies identified across the included studies, together with representative examples, techniques, and their intended biological effects, are schematically summarized in Figure 2.

**FIGURE 2:**
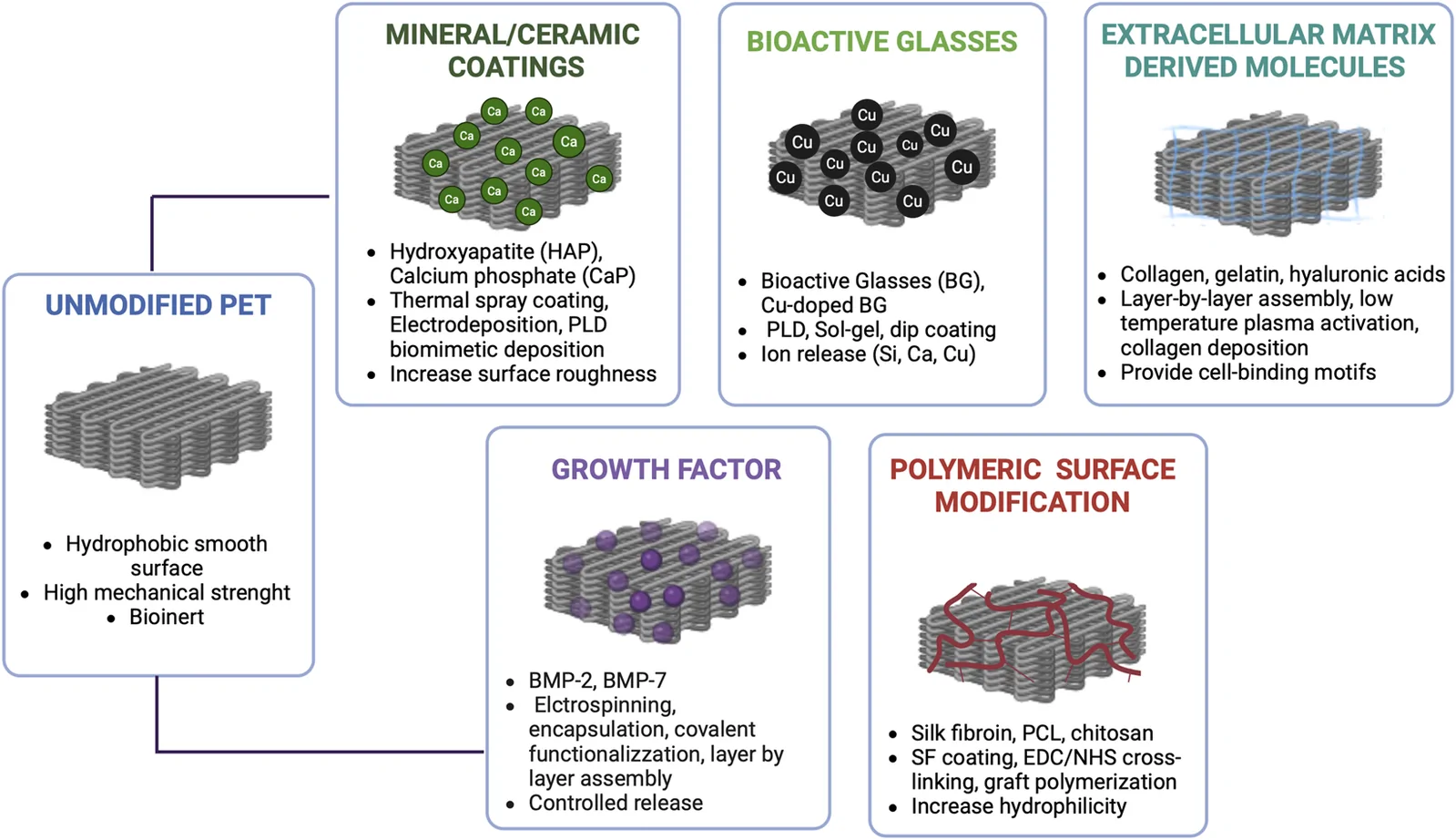
Overview of the main functionalization strategies applied to PET-based scaffolds for ACL reconstruction.

The reported approaches included physical deposition methods, chemical surface functionalization procedures, biomimetic or mineral-based coatings, and composite scaffold fabrication. Physical deposition was described in three studies (Li et al., 2012; Li et al., 2014; Wang S. et al., 2018). In particular, pulsed laser deposition (PLD) was used in two investigations to produce nanoscale bioactive glass coatings on PET scaffolds (Li et al., 2012; Li et al., 2014), while plasma spraying was applied in one only study to deposit hydroxyapatite through thermal propulsion and solidification of HAP particles onto the PET surface (Wang S. et al., 2018).

Seven studies reported different chemical surface functionalization strategies (Li et al., 2012; Vaquette et al., 2013; Wang et al., 2015; Zhang P. et al., 2016; Ai et al., 2017; Yang et al., 2019; Kang et al., 2021), including: plasma activation followed by layer-by-layer electrostatic assembly of hyaluronic acid and cationized gelatin multilayers (Li et al., 2012); ozonation-induced graft polymerization of polystyrene sodium sulfonate (Vaquette et al., 2013); transfer of chemical-vapor-deposited graphene onto PET through a poly(methyl methacrylate)-assisted process (Wang et al., 2015); low-temperature plasma activation combined with collagen coating incorporating simvastatin-loaded polycaprolactone microspheres (Zhang P. et al., 2016); covalent immobilization of silk fibroin mediated by EDC/NHS after alkali activation of PET (Ai et al., 2017); corona-discharge activation followed by hydroxypropylcellulose spray deposition (Yang et al., 2019); and formation of a polydopamine interlayer by dopamine immersion under alkaline conditions with subsequent BMP-2 immobilization (Kang et al., 2021). Biomimetic or mineral-based coatings were described in five studies (Jiang et al., 2014; Cai et al., 2018; Cai et al., 2020; Cai et al., 2021; Ma et al., 2021), including: silk fibroin coating by immersion followed by alternate soaking to induce hydroxyapatite deposition (Jiang et al., 2014); segmented silk fibroin–hydroxyapatite configuration combining EDC/NHS-mediated silk fibroin immobilization in the intra-articular region with biomimetic hydroxyapatite mineralization in the intraosseous region (Cai et al., 2018); electrochemical calcium-phosphate deposition in a Ca–P electrolyte using a two-electrode system (Cai et al., 2021); and immersion coating with hydroxyapatite (Cai et al., 2020) or strontium-substituted hydroxyapatite nanoparticles synthesized *via* microwave-hydrothermal reaction and dispersed in gelatin-containing PBS (Ma et al., 2021).

A different manufacturing configuration was reported only in one study, where the artificial ligament was constructed as a hybrid scaffold through electrospinning of polycaprolactone nanofibrous membranes, layer-by-layer incorporation of BMP-7, and subsequent lamination with PET mesh into a rolled structure (Zhang et al., 2020).

#### 3.3.3 *In vitro* outcomes

An overview of the *in vitro* cellular outcomes evaluated across the included studies is presented in Table 3 demonstrating heterogeneity in experimental design, cell types, protein expression markers and timing of assessment.

**TABLE 3:** In vitro outcomes.

Author, year	Cellular behaviour	Osteogenesis	Angiogenesis	Mineralization	Inflammatory response	Ion release
(Li et al., 2012)	Cellular proliferation At day 1, 3, 5 and 7 post-seeding: ↑ optical density (OD) in HA-CG/PET vs. PET ↑ optical density (OD) in both HA-CG/PET and PET over time Cellular viability At day 7: ↑ in both HA-CG/PET and PET	NA	At day 7 post-seeding: ↑↑ CXCL2 and CXCL8 RNA expression in HA-CG/PET vs. PET	NA	At day 7 post-seeding: ↓↓ IL-1 and TNF-α expression in HA-CG/PET vs. PET ↓↓ MMP-1 and MMP-3 expression in HA-CG/PET vs. PET	NA
(Vaquette et al., 2013)	Cellular proliferation At 24h of culture: ↑ DNA in GL vs. NGL At week 2 and 4 of culture: ↓↓ DNA in GL vs. NGL; ↓↓ DNA in GL at week 4 vs. week 2. At week 6: ↑ DNA in GL vs. NGL; From week 2 to week 6: ↓↓ DNA in GL vs. week 2; ↑↑ DNA in GL vs. week 2; ↓↓ DNA in NGL vs. week 4.	At week 2 and 4 of culture: ↑↑ ALP activity over DNA in GL vs. NGL; ↑↑ ALP activity over DNA in GL at week 4 vs. week 2. At week 6 of culture: ↑ ALP activity over DNA in GL vs. NGL	NA	From week 2 to 6 of culture: ↑↑ on GL from on week 6 vs. week 2 and 4; ↑↑ mineralization volume on GL vs. NGL. At week 6: ↓ Ca/P in GL vs. NGL; GL and NGL similar to hydroxyapatite	NA	NA
(Jiang et al., 2014)	Cellular proliferation At 24, 48, 72, 96 and 120 h of culture: ↑↑ BMSC in SFHAP/PET and SF/PET vs. PE Cellular adhesion At day 3 of culture: ↑↑ Integrin-β1 expression in SF-HAP/PET and SF/PET vs. PET	At day 14 of culture: ↑↑ Intracellular ALP in SF-HAP/PET vs. SF/PET and PET ↑↑ Extracellular ALP in SF-HAP/PET vs. SF/PET and PET	NA	NA	NA	NA
(Li et al., 2014)	Cellular proliferation At day 7 of culture: ↑↑ AKT-PET vs. PET Along time: Cells proliferated in both groups	At day 1, 3 and 7 of culture: ↑↑ ALP, RUNX2 and OPN on AKT-PET vs. PET	At day 1, 3 and 7 of culture: ↑↑VEGF on AKT-PET vs. PET	NA	NA	At day 1, 3 and 7 of culture: ↑ Si ion release in AKT-PET vs. PET Ca, P and Mg ions comparable between groups
(Wang et al., 2015)	Cellular adhesion At 24 h of culture: ↑↑ projected area per cell on G/PET vs. PET ↑↑ IOD on G/PET vs. PET At 48 h of culture: ↑ projected area per cell on G/PET vs. PET ↑↑ IOD on G/PET vs. PET Cell viability At day 1, 3, 5 and 7 of culture: ↑↑ live cell attachment on G/PET vs. PET	At day 7, 14 and 21 of culture:↑↑ RUNX2, OCN, OPN and COL1A1 on G/PET vs. PET	NA	NA	NA	NA
(Zhang P. et al., 2016)	Cellular proliferation At day 7 of culture: ↑ BMSC on SIM-COL/PET vs. COL/PET ↑↑ BMSC on SIM-COL/PET vs. PET At day 9 of culture: ↑↑ BMSC on SIM-COL/PET vs. COL/PET and PET	At day 7 of culture: ↑ ALP on SIM-COL/PET vs. COL/PET ↑↑ ALP on SIM-COL/PET vs. PET At day 14 of culture: ↑↑ ALP on SIM-COL/PET vs. COL/PET ↑↑ ALP on SIM-COL/PET vs. PET At day 1 of culture ↑↑ RUNX-2 on SIM-COL/PET vs. COL/PET and PET ↑ BMP-2 on SIM-COL/PET and COL/PET vs. PET At day 3 of culture ↑↑ OC on SIM-COL/PET vs. COL/PET and PET ↑↑ RUNX-2 on SIM-COL/PET and COL/PET vs. PET ↑↑ BMP-2 on SIM-COL/PET vs. COL/PET and PET	At day 1 of culture: ↑VEGF on SIM-COL/PET vs. COL/PET and PET At day 3 of culture: ↑ VEGF on SIM-COL/PET vs. COL/PET and PET	NA	NA	NA
(Ai et al., 2017)	Cellular proliferation At day 1 of culture: ↑ cell proliferation on SF-EDC/PET vs. PET At day 3, 5 and 7 of culture: ↑↑ cell proliferation on SF-EDC/PET vs. PET	At day 3 of culture: ↑ BMP-2, COL1 and OCN on SF-EDC/PET vs. PET At day 7 of culture: ↑↑ BMP-2, COL1 and OCN on SF-EDC/PET vs. PET	NA	NA	NA	NA
(Li et al., 2017)	NA	At day 7 of culture: ↑↑ BMP-2 and OCN in Cu-BG/PET vs. BG/PET and PET	At day 7 of culture: ↑↑ HIF-1α, VEGF and S100A10 in Cu-BG/PET vs. BG/PET and PET	NA	NA	At day 7 of culture:↑↑ Ca2+ release in Cu-BG/PET vs. BG/PET and PET↑↑ Ca2+ release in BG/PET vs. PET
(Cai et al., 2018)	Cellular proliferation At day 3 and 5 of culture: ↑↑ L929 fibroblasts on SF-HAP/PET vs. PET ↑↑ MC3T3-E1 osteoblasts on SF-HAP/PET vs. PET	At day 7 and 14 of culture: ↑↑ ALP activity on SF-HAP/PET vs. PET	NA	At day 21 and 28 of culture: ↑↑ calcium deposition on SF-HAP/PET vs. PET	NA	NA
(Wang et al., 2018a)	Cellular proliferation At day 1 and 3 of culture: ↑ cell proliferation on HAP/PET-plasma and HAP/PET-immersed vs. PET ↑cell proliferation on HAP/PET-plasma vs. HAP/PET-immersed At day 7 of culture: ↑↑ cell proliferation on HAP/PET-plasma vs. HAP/PET-immersed and PET ↑ cell proliferation on HAP/PET-immersed vs. PET At day 14 of culture: ↑↑ cell proliferation on HAP/PET-plasma and HAP/PET-immersed vs. PET ↑↑ cell proliferation on HAP/PET-plasma vs. HAP/PET-immersed	At day 3, 7 and 14 of culture ↑↑ BMP-2 and OCN on HAP/PET-plasma vs. PET	At day 3, 7 and 14 of culture: ↑↑ VEGF and eNOS on HAP/PET-plasma vs. PET ↑↑ eNOS on HAP/PET-immersed vs. PET	NA	NA	NA
(Yang et al., 2019)	Cellular proliferation At day 1 and 2: ↑BMSCs, fibroblast and RSC-346 on HPC/PET vs. PET At day 4 ↑↑fibroblast on HPC/PET vs. PET ↑ BMSCs and RSC-346 on HPC/PET vs. PET at day 6: ↑↑BMSCs and fibroblast on HPC/PET vs. PET ↑↑ RSC-346 on HPC/PET vs. PET at day 8 and 10: ↑↑ BMSCs, fibroblast and RSC-346 on HPC/PET vs. PET Cellular adhesion at day 7: ↑↑ Integrin-ß in HPC/PET vs. PET	At day 7: ↑↑ BMP-2 and BMP-7 in HPC/PET vs. PET At day 5, 7, 10 and 14 of culture: ↑↑ ALP and OPN in HPC/PET vs. PET	NA	NA	NA	NA
(Cai et al., 2020)	Cellular proliferation At day 3 and 5 of culture: ↑↑ CCK-8 in HAP-PET vs. PET Cellular adhesion At day 1 and 5 of culture: ↑↑ cell spreading area on HAP-PET vs. PET	At day 7 and 14 of culture: ↑↑ ALP IN HAP-PET vs. PET ↑↑ COL1, OCN and OPN in HAP-PET vs. PET ↑↑ COL 1, OCN and OPN in HAP-PET vs. PET↑↑ p-ERK/ERK ratio in HAP-PET vs. PET	NA	At day 21 and 28 of culture: ↑↑ Alizarin Red staining/mineral nodule quantification on HAP-PET vs. PET	NA	NA
(Zhang et al., 2020)	Cellular proliferation at day 1 and 3 of culture:↑ BMSCs in BMP-7-PCL/PET and PCL/PET vs. PET At day 5 of culture: ↑↑ BMSCs in BMP-7-PCL/PET vs. PET ↑ BMSCs in BMP-7-PCL/PET vs. PCL/PET At day 7 of culture: ↑↑ BMSCs in BMP-7-PCL/PET vs. PCL/PET and PET	At day 7 and 14 of culture: ↑↑ ALP in BMP-7-PCL/PET vs. PCL/PET and PET ↑↑ ALP in PCL/PET vs. PET At day 7 of culture :↑↑ RUNX2 and BMP2 in BMP-7-PCL/PET vs. PCL/PET and PET ↑ OCN in BMP-7-PCL/PET vs. PCL/PET and PET ↑ OPN in PCL/PET vs. BMP-7-PCL/PET and PET at day 14 of culture: ↑↑ RUNX2, OPN, BMP2 and OCN in BMP-7-PCL/PET vs. PCL/PET and PET ↑ RUNX2, OPN and OCN in PCL/PET vs. PET	NA	at day 30 of culture: ↑ Calcium in BMP-7-PCL/PET and PCL/PET vs. PET group ↑↑ Calcium BMP-7-PCL/PET vs. PCL/PET	NA	NA
(Cai et al., 2021)	Cellular proliferation At day 1: no significant differences between BM-CaP/PET, ED-CaP/PET and PET At day 3 and 5: ↑↑ cells proliferation in BM-CaP/PET and ED-CaP/PET vs. PET Cellular viability At day 1: no significant differences between BM-CaP/PET, ED-CaP/PET and PET At day 5: ↑↑ cells are over total area in BM-CaP/PET and ED-CaP/PET vs. PET	At day 7 of culture: ↑↑ ALP, COL1, OCN and OPN in ED-CaP/PET and BM-CaP/PET vs. PET ↑↑ ALP, COL1 and OPN in ED-CaP/PET vs. BM-CaP/PET At day 14 of culture: ↑↑ ALP, COL1, OCN and OPN in ED-CaP/PET and BM-CaP/PET vs. PET ↑↑ ALP, COL1, OCN and OPN in ED-CaP/PET vs. BM-CaP/PET	NA	NA	NA	NA
(Kang et al., 2021)	Cellular proliferation At day 1: ↑ BMSCs in 500-PDA/PET and PDA/PET vs. PET At day 3 and 7: ↑↑ BMSCs in 500B-PDA/PET vs. PET ↑ BMSCs in PDA/PET vs. PET	At day 7 of culture: ↑↑ RUNX2 and ALP in 500B-PDA/PET vs. PET ↑ OPN and OCN in 500B-PDA/PET and PDA/PET vs. PET ↑ RUNX2 and ALP in PDA/PET vs. PET At day 14 of culture: ↑↑ RUNX2, ALP, OPN and OCN in 500B-PDA/PET and PDA/PET vs. PET	NA	At day 21 of culture: ↑↑ calcium nodule sedimentation in 500B-PDA/PET vs. PET ↑ calcium nodule sedimentation in PDA/PET vs. PET	NA	NA
(Ma et al., 2021)	Cellular proliferation At day 1 and 3 of culture: ↑ BMSCs in HAP-PET and 2SrHAP-PET vs. PET At day 5 of culture: ↑↑ BMSCs in 2SrHAP-PET vs. PET ↑ BMSCs in HAP-PET vs. PET	At day 7 of culture: ↑↑ ALP, COL1, OCN, OPN in HAP-PET and 2SrHAP-PET vs. PET ↑↑ BMP-2, OCN, COL1 in 2SrHAP-PET vs. PET and HAP-PET ↑↑ ALP activity in 2SrHAP-PET vs. PET and HAP-PET ↑ ALP activity in HAP-PET vs. PET	At day 7 of culture: ↑↑ VEGF in 2SrHAP-PET vs. PET and HAP-PET ↑↑ VEGF in HAP-PET vs. PET	At day 14 of culture: ↑ in 2SrHAP-PET vs. PET and HAP-PET	NA	NA
(Wei et al., 2026)	Cellular viability At day 3, 5 and 14 of culture: ↑↑ BP/SF-PET vs. PET at day 3, 5 and 14	At day 7 and 14 of culture: ↑↑ COL1, OPN and OCN on BP/SF-PET vs. PET	NA	At day 21 and 28 of culture: ↑↑ calcium nodule deposition on BP/SF-PET vs. PET	NA	NA

ACL, anterior cruciate ligament; AKT, akermanite; ALP, alkaline phosphatase; BG, bioactive glass; BM-CaP, biomineralized calcium phosphate; BMP-2, Bone Morphogenetic Protein-2; BMP-7, Bone Morphogenetic Protein-7; BMP-7-PCL/PET, BMP-7-loaded PCL/PET, hybrid scaffold; BP, black phosphorous; BP/SF-PET, Black phosphorous and silk fibroin-coated PET; BMSC, bone marrow stromal cell; CaP, calcium phosphate; CG, cationized gelatin; COL, collagen; COL1/COL1A1, Collagen type I alpha 1; Cu-BG, Copper-containing Bioactive Glass; CXCL2, C-X-C motif chemokine ligand 2; CXCL8, C-X-C motif chemokine ligand 8; DNA, deoxyribonucleic acid; EDC, 1-Ethyl-3-(3-dimethylaminopropyl) carbodiimide; EDS, Energy-Dispersive Spectroscopy; ED-CaP, electrodeposited calcium phosphate; eNOS, endothelial nitric oxide synthase; ERK, Extracellular signal-regualted Kinase; GL, grafted ligament; G/PET, Graphene-coated PET; HA, hyaluronic acid; HA-CG, Hyaluronic Acid-Cationized Gelatin; HAP, hydroxyapatite; HAP/PET-immersed, immersion-coated hydroxyapatite PET; HAP/PET-plasma, plasma spray-coated hydroxyapatite PET; HIF-1α, Hypoxia-Inducible Factor-1, alpha; HPC, hydroxypropylcellulose; IL-1, Interleukin-1; Integrin-β1, Integrin beta-1; IOD, integrated optical density; L929, murine fibroblast cell line; LbL, Layer-by-Layer; MMP-1, Matrix Metalloproteinase-1; MMP-3, Matrix Metalloproteinase-3; MC3T3-E1, murine preosteoblast cell line; NHS, N-Hydroxysuccinimide; NGL, non-grafted ligament; OD, optical density; OCN, osteocalcin; OPN, osteopontin; PCL, polycaprolactone; PDA, polydopamine; PET, polyethylene terephthalate; PLD, pulse laser deposition; p-ERK, phosphorylated Extracellular signal-regualted Kinase; RSC-346, rabbit Schwann cell line 346; RUNX2, Runt-related transcription factor 2; S100A10, protein-binding Ca2+; SEM, scanning electron microscopy; SF, silk fibroin; SF-EDC/PET, Silk fibroin-coated PET, stabilized with EDC/NHS, crosslinking; SF-HAP, Silk Fibroin-Hydroxyapatite; SIM, simvastatin; SIM-COL/PET, Simvastatin-collagen-coated PET; SrHAP, Strontium-substituted Hydroxyapatite; TEM, transmission electron microscopy; TGA, thermogravimetric analysis; TNF-α, tumor necrosis factor alpha; VEGF, vascular endothelial growth factor; XPS, X-ray Photoelectron Spectroscopy; XRD, X-ray Diffraction; ↓ lower (non significant); ↓↓ significantly lower; ↑ higher (non significant); ↑↑ significantly higher.

##### 3.3.3.1 Cellular activity

Cellular activity was evaluated in 16 studies (Li et al., 2012; Vaquette et al., 2013; Jiang et al., 2014; Li et al., 2014; Wang et al., 2015; Zhang P. et al., 2016; Ai et al., 2017; Cai et al., 2018; Wang S. et al., 2018; Yang et al., 2019; Cai et al., 2020; Zhang et al., 2020; Cai et al., 2021; Kang et al., 2021; Ma et al., 2021; Wei et al., 2026).

Assessments were performed at early and intermediate time points, ranging from 24 h (Li et al., 2012; Vaquette et al., 2013; Jiang et al., 2014; Wang et al., 2015; Ai et al., 2017; Wang S. et al., 2018; Yang et al., 2019; Zhang et al., 2020; Cai et al., 2021; Kang et al., 2021; Ma et al., 2021; Wei et al., 2026)to 14 days of culture (Wang S. et al., 2018), with one study extending the evaluation up to 6 weeks (Vaquette et al., 2013).

Cellular behaviour was evaluated in 16 studies (Li et al., 2012; Vaquette et al., 2013; Jiang et al., 2014; Li et al., 2014; Wang et al., 2015; Zhang P. et al., 2016; Ai et al., 2017; Cai et al., 2018; Wang S. et al., 2018; Yang et al., 2019; Cai et al., 2020; Zhang et al., 2020; Cai et al., 2021; Kang et al., 2021; Ma et al., 2021; Wei et al., 2026). Assessments were conducted across multiple time points, ranging from 24 h (Vaquette et al., 2013; Jiang et al., 2014; Wang et al., 2015) to up to 6 weeks of culture (Vaquette et al., 2013). Overall, functionalized grafts demonstrated improved cellular response compared with unmodified PET controls, including enhanced proliferation, adhesion, and viability.

Cellular proliferation was the most frequently investigated parameter and was assessed in 14 studies (Li et al., 2012; Vaquette et al., 2013; Jiang et al., 2014; Li et al., 2014; Zhang P. et al., 2016; Ai et al., 2017; Cai et al., 2018; Wang S. et al., 2018; Yang et al., 2019; Cai et al., 2020; Zhang et al., 2020; Cai et al., 2021; Kang et al., 2021; Ma et al., 2021). In most of these, functionalized grafts showed higher or significantly higher proliferation rates compared with PET controls, with increases observed as early as 24 h/day (Jiang et al., 2014; Ai et al., 2017; Wang S. et al., 2018; Yang et al., 2019; Zhang et al., 2020; Kang et al., 2021; Ma et al., 2021), and maintained over time up to day 14 and beyond (Wang S. et al., 2018). Several studies also reported progressive increases over time within the same group (Li et al., 2012; Li et al., 2014). In some cases, functionalized grafts showed superior performance not only compared with PET but also relative to alternative coatings (Zhang P. et al., 2016; Zhang et al., 2020).

An exception was reported by Vaquette et al. (2013), where DNA content showed variable trends over time, with initial increases followed by decreases and subsequent recovery at later time points.

Cellular adhesion was evaluated in three studies (Jiang et al., 2014; Wang et al., 2015; Yang et al., 2019).

All three reported increased adhesion-related parameters in functionalized grafts, including higher integrin expression (Jiang et al., 2014; Yang et al., 2019) and greater cell spreading and attachment (Wang et al., 2015), particularly at early time points (24–72 h).

Cell viability was assessed in four studies (Li et al., 2012; Wang et al., 2015; Cai et al., 2021; Wei et al., 2026).

The overall trend favored Functionalized grafts, which showed improved or significantly improved viability compared with PET controls, particularly at later time points such as day 5–14 (Cai et al., 2021; Wei et al., 2026), although no significant differences were observed at very early stages in some cases (Cai et al., 2021).

##### 3.3.3.2 Angiogenesis and inflammation

Angiogenesis was evaluated in six studies (Li et al., 2012; Li et al., 2014; Zhang P. et al., 2016; Li et al., 2017; Wang S. et al., 2018; Ma et al., 2021).

Assessments were performed at early and intermediate time points, ranging from 1 day to 14 days of culture or post-seeding. The overall trend across these studies favored functionalized grafts, which showed increased angiogenetic marker expression or activity compared with unmodified PET controls.

Five studies reported a significantly increased expression of Vascular Endothelial Growth Factor (VEGF) in functionalized grafts compared with PET controls (Li et al., 2014; Zhang P. et al., 2016; Li et al., 2017; Wang S. et al., 2018; Ma et al., 2021). Upregulation was observed starting from the day 1 (Li et al., 2014; Zhang P. et al., 2016) and persisted at later time points up to day 14 (Wang S. et al., 2018). Additional angiogenesis-related markers were investigated in three studies. At day 7, Li et al. (2017) reported significantly higher expression of HIF-1α and S100A10 in Cu-BG/PET compared with BG/PET and PET, while Li et al. (2012) observed significantly increased RNA expression of the chemokines CXCL2 and CXCL8 in HA-CG/PET when compared to PET. Wang S. et al. (2018) reported significantly higher eNOS expression in both HAP/PET-plasma and HAP/PET-immersed grafts when compared to PET controls at days 3, 7 and 14.

Inflammatory response was evaluated only by Li et al. (2012) who reported significantly lower expression of the pro-inflammatory cytokines IL-1 and TNF-α, as well as significantly lower expression of MMP-1 and MMP-3, in functionalized grafts (HA-CG/PET) compared with unmodified PET at 7 days post seeding.

##### 3.3.3.3 Osteogenesis

Osteogenesis was evaluated in 16 studies (Li et al., 2012; Vaquette et al., 2013; Jiang et al., 2014; Li et al., 2014; Wang et al., 2015; Zhang P. et al., 2016; Ai et al., 2017; Cai et al., 2018; Wang S. et al., 2018; Yang et al., 2019; Cai et al., 2020; Zhang et al., 2020; Cai et al., 2021; Kang et al., 2021; Ma et al., 2021; Wei et al., 2026).

Assessments were performed over a broad range of time points, from 1 day (Li et al., 2014; Zhang P. et al., 2016) to 6 weeks of culture (Vaquette et al., 2013).

The overall trend favored functionalized grafts, which showed increased osteogenic marker expression or activity compared with unmodified PET controls in the large majority of studies.

ALP was the most frequently reported osteogenic marker and was assessed in 11 articles (Vaquette et al., 2013; Jiang et al., 2014; Li et al., 2014; Zhang P. et al., 2016; Cai et al., 2018; Yang et al., 2019; Cai et al., 2020; Zhang et al., 2020; Cai et al., 2021; Kang et al., 2021; Ma et al., 2021). Its upregulation was observed as early as day 1 and 3 and persisted at later time points up to week 6 (Vaquette et al., 2013). In nearly all cases, functionalized grafts demonstrated higher or significantly higher ALP levels compared with PET controls (Vaquette et al., 2013; Jiang et al., 2014; Li et al., 2014; Zhang P. et al., 2016; Cai et al., 2018; Yang et al., 2019; Cai et al., 2020; Zhang et al., 2020; Cai et al., 2021; Kang et al., 2021; Ma et al., 2021), with some studies also reporting higher ALP values compared with alternative coated grafts (Zhang P. et al., 2016; Zhang et al., 2020; Cai et al., 2021; Ma et al., 2021).

RUNX2 upregulation was reported in five studies (Li et al., 2014; Wang et al., 2015; Zhang P. et al., 2016; Zhang et al., 2020; Kang et al., 2021), with increased expression detected from day 1 and day 3 (Li et al., 2014; Zhang P. et al., 2016) and maintained up to day 14 (Zhang et al., 2020; Kang et al., 2021).

BMP-2 upregulation was observed in seven studies (Zhang P. et al., 2016; Ai et al., 2017; Li et al., 2017; Wang S. et al., 2018; Yang et al., 2019; Zhang et al., 2020; Ma et al., 2021), most frequently at day 7 of culture (Ai et al., 2017; Li et al., 2017; Wang S. et al., 2018; Yang et al., 2019; Ma et al., 2021).

Late osteogenic markers, including osteocalcin (OCN), osteopontin (OPN) and collage type I (COL1), were significantly upregulated in functionalized grafts compared with PET in 13 studies (Li et al., 2014; Wang et al., 2015; Zhang P. et al., 2016; Ai et al., 2017; Li et al., 2017; Wang S. et al., 2018; Yang et al., 2019; Cai et al., 2020; Zhang et al., 2020; Cai et al., 2021; Kang et al., 2021; Ma et al., 2021; Wei et al., 2026).

Cai et al. (2020) additionally reported a significant increase in the p-ERK/ERK ratio in HAP/PET vs. PET at days 7 and 14.

##### 3.3.3.4 Mineralization

Mineralization was evaluated in seven studies (Vaquette et al., 2013; Cai et al., 2018; Cai et al., 2020; Zhang et al., 2020; Kang et al., 2021; Ma et al., 2021; Wei et al., 2026).

The timing of assessment ranged from day 14 to day 30 of culture, with one study extending the evaluation up to week 6 (Vaquette et al., 2013).

Six of these studies reported higher or significantly higher calcium deposition or mineralized tissue formation in functionalized grafts compared with PET controls (Cai et al., 2018; Cai et al., 2020; Zhang et al., 2020; Kang et al., 2021; Ma et al., 2021; Wei et al., 2026).

Vaquette et al. (2013) quantified mineralization volume over time and reported significantly higher mineralization in grafted ligaments (GL) compared with non-grafted ligaments (NGL), with a progressive increase from week 2 to week 6. In the same study, the Ca/P ratio measured at week 6 was lower in GL compared with NGL, while remaining comparable to hydroxyapatite in both groups.

In studies directly comparing different functionalization strategies, greater calcium deposition was observed in more highly functionalized grafts, such as BMP-7-PCL/PET compared with PCL/PET (Zhang et al., 2020) and 500B-PDA/PET compared with PDA/PET (Kang et al., 2021).

##### 3.3.3.5 Ion release

Ion release was evaluated in two studies (Li et al., 2014; Li et al., 2017).

Li et al. (2017) assessed calcium ion release and reported significantly higher Ca^2+^ release from Cu-BG/PET compared with both BG/PET and PET, and significantly higher Ca^2+^ release from BG/PET compared with PET at day 7 of culture. Li et al. (2014) evaluated silicon ion release and observed higher Si ion release from AKT-PET compared with PET at days 1, 3, and 7. In the same study, calcium, phosphorus, and magnesium ion concentrations were comparable between groups.

#### 3.3.4 Animal models

An overview of the animal model employed across the included studies are presented in Table 4.

**TABLE 4:** Animal model and mechanical properties outcomes.

Author, year	Animal model	Mechanical properties Pre-implant and/or post-implant
(Li et al., 2012)	1) 30 New Zealand rabbits mature male, mean weight 3.2 ± 0.9 kg n = 15 rabbits per group Interventional group: HA-CG LBL-coated PET artificial ligament (HA-CG/PET) Control group: Uncoated PET artificial ligament (PET) 2) 6 GuangXi pigs mature male, mean weight 43.4 ± 8.5 kg n = 3 GuangXi pigs per group Interventional group: HA-CG LBL-coated PET artificial ligament (HA-CG/PET) Control group: Uncoated PET artificial ligament (PET)	Tensile strength pre implantation PET = 165.9 MPa HA-CG/PET = 164.47 MPa no significant differences between groups Tensile strain pre implantation PET = 221.05% HA-CG/PET = 218.422% no significant differences between groups
(Vaquette et al., 2013)	51 Pré-Alpes sheep female, 2 years old n = 28 Grafted Ligament; n = 23 Non-Grafted Ligament; Interventional group: grafted LARS™ PET (GL) Control group: non-grafted LARS™ PET (NGL)	Ultimate failure load At month 3 post implantation ↑ GL (188 ± 52 N) vs. NGL (144 ± 69 N) similar failure modes between groups At month 12 post implantation ↑ GL (322 ± 170 N) vs. NGL (260 ± 126 N) normalized failure load higher for GL (32%) vs. NGL (21%) similar failure modes between groups
(Jiang et al., 2014)	72 New Zealand rabbits mature male, mean weight 2.8 ± 0.5 kg n = 24 per group, randomly divided Interventional group: SF/PET and SF-HAP/PET Control group: PET	Ultimate failure load at week 4 post implantation ↑ SF-HAP/PET (44.4 ± 8.3 N) vs. SF/PET (40.6 ± 5.2 N) and PET (38.8 ± 5.1 N) at week 8 post implantation ↑↑ SF-HAP/PET (91.0 ± 5.39 N) vs. SF/PET (62.8 ± 5.59 N) and PET (56.6 ± 5.37 N) Stiffness at week 4 post implantation ↑ SF-HAP/PET (5.2 ± 1.5 N/mm) vs. SF/PET (4.8 ± 1.2 N/mm) and PET (4.0 ± 1.7 N/mm) at week 8 post implantation ↑↑ SF-HAP/PET (8.1 ± 1.8 N/mm) vs. SF/PET (6.0 ± 1.0N/mm) and PET (5.2 ± 0.8 N/mm)
(Li et al., 2014)	30 New Zealand rabbits mature male, mean weight 2.7 ± 0.6 kg n = 60 knee total, 30 for each group Interventional group: AKT/PET (right knee) Control group: PET (left knee)	Ultimate load failure (Pull out load) at week 2 post implantation ↑ AKT-PET (35.2 N) vs. PET (33.5 N) at week 4 post implantation ↑↑ AKT-PET (84.8 N) vs. PET (52.5 N) AKT-PET group was 61% higher than that of the PET group at week 8 post implantation ↑↑ AKT-PET (98.4 N) vs. PET (59.2 N) AKT-PET group was 67% higher than that of the PET group
(Wang et al., 2015)	32 New Zealand rabbits male, 12 weeks old, average weight 3.0 ± 0.4 kg n = 64 knees total, 32 per group Interventional group: G/PET Control group: PET (contralateral limb)	Ultimate failure load At week 4 post implantation ↑↑ G/PET (46.2 N) vs. PET (26.2 N) At week 8 post implantation ↑↑ G/PET (63.9 N) vs. PET (45.5 N) At week 12 post implantation ↑↑ G/PET (80.4 N) vs. PET (52.3 N) G/PET reached ∼70% of native ACL strength Stiffness At week 4 post implantation ↑↑ G/PET (16.4 N/mm) vs. PET (7.2 N/mm) At week 8 post implantation ↑↑ G/PET (26.7 N/mm) vs. PET (17.6 N/mm) At week 12 post implantation ↑↑ G/PET (34.8 N/mm) vs. PET (20.8 N/mm) G/PET reached ∼46% of native ACL stiffness
(Zhang et al., 2016b)	36 New Zealand White rabbits adult, average weight of 2.5–3.5 kg n = 12 rabbit for each group Interventional group: SIM-COL/PET, COL/PET Control group: PET	Ultimate load failure At 4 weeks post implantation ↑ SIM-COL/PET (48.6 N) vs. COL/PET (31.6 N), and PET (26.8 N), At 8 weeks post implantation ↑↑ SIM-COL/PET (86.4 N) vs. COL/PET (53.4 N) and PET (51.8 N) Stiffness At 4 weeks post implantation ↑ SIM-COL/PET (10 N/mm) vs. COL/PET (12 N/mm) and PET (9.2 N/mm) At 8 weeks post implantation ↑↑ SIM-COL/PET (26.1 N/mm) vs. COL/PET (17.5 N/mm) and PET (16.2 N/mm)
(Ai et al., 2017)	46 New Zealand rabbits mature male, average weight 2.8 ± 0.5 Kg n = 23 per group, randomly divided Interventional group: SF-EDC/PET Control group: PET	Ultimate load failure At week 6 post implantation ↑ SF-EDC/PET (46.6 ± 4.1 N) vs. PET (46.0 ± 4.2 N) At week 12 post implantation ↑ SF-EDC/PET (70.1 ± 6.5 N) vs. PET (66.0 ± 6.1 N) Stiffness At week 6 post implantation ↑ SF-EDC/PET (4.8 ± 0.6 N/mm) vs. PET (4.4 ± 0.3 N/mm) At week 12 post implantation ↑ SF-EDC/PET (6.5 ± 0.8 N/mm) vs. PET (6.2 ± 0.8 N/mm)
(Li et al., 2017)	24 goats healthy skeletally mature female n = 8 goats for each group Interventional groups: BG/PET and Cu-BG/PET Control group: PET	Ultimate failure load (pull-out load) at week 12 post implantation ↑↑ Cu-BG/PET (182.7 N) and BG/PET (129.0 N) vs. PET (88.4 N) ↑ Cu-BG/PET (89.03 N) vs. BG/PET (89.03 N Cu-BG/PET (182.7 N) and BG/PET (129.0 N))
(Cai et al., 2018)	24 beagle dogs male, healthy skeletally mature n = 24 dogs for each group n = 48 knee total, 24 for each group Interventional group: SF-HAP/PET Control group: PET	Ultimate load failure at week 6 post implantation ↑↑ in SF-HAP/PET (291 ± 27.3 N) vs. PET (169.8 ± 19.4 N) ↓ in SF-HAP/PET vs. native ACL at week 12 post implantation ↑↑ in SF-HAP/PET (439.2 ± 27.3 N) vs. PET (198.3 ± 17.8 N) ↓ in SF-HAP/PET vs. native ACL Stiffness at week 6 post implantation ↑↑ in SF-HAP/PET (64.6 ± 3.1 N/mm) vs. PET (39.3 ± 4.2 N/mm) ↓ in SF-HAP/PET vs. native ACL at week 12 post implantation ↑↑ in SF-HAP/PET (99.4 ± 3.9 N/mm) vs. PET (45.1 ± 6.0 N/mm) ↓ in SF-HAP/PET vs. native ACL
(Wang et al., 2018a)	72 New Zealand rabbits average weight of 2.9 ± 0.2 kg n = 24 per group, randomly divided Interventional group: HAP/PET-plasma and HAP/PET-immersed Control group: PET	Ultimate load failure At week 4 post implantation ↑ HAP/PET-plasma (35.7 N) and HAP/PET-immersed (43.3 N) vs. PET (43.7 N) At week 8 post implantation ↑↑ HAP/PET-plasma (70.7 N) and HAP/PET-immersed (65.1 N) vs. PET (49.9 N) At week 12 post implantation PET = 52.6 N ↑↑ HAP/PET-plasma (78.0 N) and HAP/PET-immersed (72.3 N) vs. PET (52.6 N) Stiffness At week 4 post implantation ↑↑ HAP/PET-plasma (23.4 N/mm) and HAP/PET-immersed (21.1 N/mm) vs. PET (14.5 N/mm) At week 8 post implantation ↑↑ HAP/PET-plasma (26.4 N/mm) and HAP/PET-immersed (26.9 N/mm) vs. PET (18.9 N/mm) At week 12 post implantation HAP/PET-plasma = 31.2 N/mm HAP/PET-immersed = 29.7 N/mm PET = 20.2 N/mm ↑↑ HAP/PET-plasma and HAP/PET-immersed vs. PET
(Yang et al., 2019)	24 beagle dogs male, healthy, average weight 12.40 ± 1.58 kg n = 12 dogs for each group Interventional group: HPC/PET Control group: PET	Ultimate load failure at week 4 post implant ↑↑ in HPC/PET (325.0 ± 32.6 N) vs. PET (142.0 ± 5.3N) at week 16 post implant ↑↑ in HPC/PET (502.3 ± 20.0 N) vs. PET (182.7 ± 14.5 N) Stiffness at week 4 post implant ↑ in HPC/PET (23.0 ± 5.0 N/mm) vs. PET (18.3 ± 3.5 N/mm) at week 16 post implant ↑↑ in HPC/PET (46.7 ± 7.8 N/mm) vs. PET (27.3 ± 4.5 N/mm)
(Cai et al., 2020)	48 New Zealand white rabbits male, mature, weight 2.5–3.4 kg n = 24 rabbits for each group Interventional group: HAP/PET Control group: PET	Ultimate failure load at week 6 post implantation ↑ HAP/PET (38.1 ± 5.6 N) vs. PET (34.5 ± 4.4 N) at week 12 post implantation ↑↑ HAP/PET (76.4 ± 6.3 N) vs. PET (52.1 ± 6.3 N) Stiffness at week 6 post implantation ↑ HAP/PET (7.5 ± 1.1 N/mm) vs. PET (7.3 ± 1.3 N/mm) at week 12 post implantation ↑↑ HAP/PET (14.9 ± 3.1 N/mm) vs. PET (10.1 ± 1.6 N/mm)
(Zhang et al., 2020)	72 New Zealand white rabbits average weight of 2.5–3.5 kg n = 24 rabbits for each group Interventional group: PCL/PET, BMP-7/PCL/PET Control group: PET	Ultimate load failure at week 6 post implantation ↑↑ in BMP-7-PCL/PET (50.1 N) vs. PCL/PET (45.9 N) and PET (42.8 N) ↑ in PCL/PET (45.9 N) vs. PET (42.8 N) at week 12 post implantation ↑↑ in BMP-7-PCL/PET (101.7 N) vs. PCL/PET (74.1 N) and PET (63.1 N) ↑ in PCL/PET (74.1 N) vs. PET (63.1 N) Stiffness at week 6 post implantation BMP-7-PCL/PET = 16.05 N/mm PCL/PET = 14.01 N/mm PET = 10.57 N/mm ↑↑ in BMP-7-PCL/PET vs. PET ↑ in BMP-7-PCL/PET vs. PCL/PET ↑ in PCL/PET vs. PET at week 12 post implantation BMP-7-PCL/PET = 28.66 N/mm PCL/PET = 16.82 N/mm PET = 14.01 N/mm ↑↑ in BMP-7-PCL/PET vs. PCL/PET and PET ↑ in PCL/PET vs. PET
(Cai et al., 2021)	60 New Zealand rabbits mean weight of 3.0 ± 0.5 kg n = 20 rabbits per group Interventional group: BM-CaP/PET, ED-CaP/PET Control group: PET	Ultimate load failure at week 6 post implantation ↑↑ in ED-CaP/PET (40.4 ± 10.5 N) and BM-CaP/PET (39.0 ± 6.3 N) vs. PET (24.3 ± 6.7 N) at week 12 post implantation ↑↑ in ED-CaP/PET (93.9 ± 6.7 N) vs. BM-CaP/PET (76.4 ± 6.3 N) Stiffness at week 6 post implantation ↑ in ED-CaP/PET (10.0 N/mm) vs. BM-CaP/PET (8.9 N/mm) and PET (6.6 N/mm) ↑ in BM-CaP/PET (8.9 N/mm) and PET (6.6 N/mm) at week 12 post implantation ↑↑ in ED-CaP/PET (23.1 ± 3.9 N/mm) vs. BM-CaP/PET (15.8 ± 2.5 N/mm) and PET (10.2 ± 2.3 N/mm) ↑↑ in BM-CaP/PET (15.8 ± 2.5 N/mm) and PET (10.2 ± 2.3 N/mm)
(Kang et al., 2021)	15 New Zealand rabbits male, 10 weeks old, average weight 2.7 ± 0.3 kg n = 5 rabbits per group Interventional group: 500B-PDA/PET, PDA/PET Control group: PET	Ultimate load failure at week 8 post implantation ↑↑ in 500B-PDA/PET (79.9 ± 6.5 N) and PDA/PET (58.0 ± 4.9 N) vs. PET (44.3 ± 4.0 N)
(Ma et al., 2021)	54 New Zealand rabbits n = 18 per groups Interventional group: HAP/PET, 2SrHAP/PET Control group: PET	Ultimate load failure at week 4 post implantation ↑ in 2SrHAP/PET (76.5 ± 14.5 N) and HAP/PET (53.3 ± 10.7 N) vs. PET (47.0 ± 8.5 N) ↑ in 2SrHAP/PET (76.5 ± 14.5 N) vsHAP/PET (53.3 ± 10.7 N) At week 8 post implantation ↑ in 2SrHAP/PET (94.8 ± 20.8 N) and HAP/PET (80.6 ± 14.6 N) vs. PET (57.0 ± 13.0N) ↑ in 2SrHAP/PET (94.8 ± 20.8 N) vsHAP/PET (80.6 ± 14.6 N) at week 12 post implantation ↑↑ in 2SrHAP/PET (121.5 ± 25.2 N) vs. PET (48.3 ± 16.1 N) ↑ in 2SrHAP/PET (121.5 ± 25.2 N) vs. HA/PET (85.9 ± 18.3 N) ↑ in HAP/PET (85.9 ± 18.3N) vs. PET (48.3 ± 16.1 N)
(Wei et al., 2026)	30 New Zealand white rabbit male, mature, weight 2.8–3.5 kg n = 15 per group (*via* computer-generated random number table) Interventional group: BP-SF/PET Control group: PET	Ultimate load failure at week 6 post implantation ↑ BP-SF/PET (40.8 ± 4.0 N) vs. PET (37.1 ± 3.5 N) at week 12 post implantation ↑↑ BP-SF/PET (73.7 ± 4.2 N) vs. PET (52.9 ± 6.3 N) Stiffness at week 6 post implantation ↑ BP-SF/PET (14.8 ± 4.9 N/mm) vs. PET (12.1 ± 3.8 N/mm) at week 12 post implantation ↑↑ BP-SF/PET (23.5 ± 2.7 N/mm) vs. PET (18.5 ± 4.5N/mm)

2SrHAP/PET: PET, coated with 2% strontium-substituted hydroxyapatite; 500B-PDA/PET: BMP-2, immobilized PDA-coated PET; ACL: anterior cruciate ligament; AKT: akermanite; AKT-PET: Akermanite-coated PET; BG: bioactive glass; BM-CaP/PET: Biomineralized Calcium Phosphate-coated PET; BMP-2: Bone Morphogenetic Protein-2; BMP-7: Bone Morphogenetic Protein-7; BMP-7/PCL/PET: BMP-7-loaded PCL/PET, hybrid scaffold; BP: black phosphorous; BP-SF/PET: Black phosphorous and silk fibroin-coated PET; CaP: calcium phosphate; CG: cationized gelatin; COL: collagen; Cu-BG: Copper-containing Bioactive Glass; ED-CaP/PET: Electrodeposited Calcium Phosphate-coated PET; EDC: 1-Ethyl-3-(3-dimethylaminopropyl) carbodiimide; EDS: Energy-Dispersive Spectroscopy; G/PET: Graphene-coated PET; GL: grafted ligament; HA: hyaluronic acid; HA-CG/PET: Hyaluronic Acid-Cationized Gelatin-coated PET; HAP: hydroxyapatite; HAP/PET-immersed: immersion-coated hydroxyapatite PET; HAP/PET-plasma: plasma spray-coated hydroxyapatite PET; HPC: hydroxypropylcellulose; LARS™: ligament advanced reinforcement system; LbL: Layer-by-Layer; NHS: N-Hydroxysuccinimide; NGL: Non-Grafted Ligament; PCL: polycaprolactone; PDA: polydopamine; PET: polyethylene terephthalate; SF: silk fibroin; SF-EDC/PET: Silk fibroin-coated PET, stabilized with EDC/NHS, crosslinking; SF-HAP/PET: Silk Fibroin/Hydroxyapatite-coated PET; SIM: simvastatin; SIM-COL/PET: Simvastatin-collagen-coated PET; SrHAP: Strontium-substituted Hydroxyapatite; TGA: thermogravimetric analysis; TEM: transmission electron microscopy; XPS: X-ray Photoelectron Spectroscopy; XRD: X-ray Diffraction; ↓ lower (non significant); ↓↓ significantly lower; ↑ higher (non significant); ↑↑ significantly higher.

A total of 726 animals were included for *in vivo* analysis: 24 goats (3.31%) (Li et al., 2017), 51 Pré-Alpes sheep (7.02%) (Vaquette et al., 2013), 6 GuangXi pigs (0.83%) (Li et al., 2012), 48 beagle dogs (6.61%) (Cai et al., 2018; Yang et al., 2019), and 597 New Zealand rabbits (82.23%) (Li et al., 2012; Jiang et al., 2014; Li et al., 2014; Wang et al., 2015; Zhang P. et al., 2016; Ai et al., 2017; Wang S. et al., 2018; Cai et al., 2020; Zhang et al., 2020; Cai et al., 2021; Kang et al., 2021; Ma et al., 2021; Wei et al., 2026). All included studies primarily employed a single animal species; one study only (Li et al., 2012) used two different species (New Zealand rabbits and GuangXi pigs), while maintaining separate experimental groups for each species.

In three studies (Li et al., 2014; Wang et al., 2015; Cai et al., 2018), the interventional graft was implanted in one knee and the control PET graft in the contralateral knee of the same animal. In the remaining 14 studies (Li et al., 2012; Vaquette et al., 2013; Jiang et al., 2014; Zhang P. et al., 2016; Ai et al., 2017; Li et al., 2017; Wang S. et al., 2018; Yang et al., 2019; Cai et al., 2020; Zhang et al., 2020; Cai et al., 2021; Kang et al., 2021; Ma et al., 2021; Wei et al., 2026), interventional and control grafts were implanted in different animals, always of the same species.

Animal age was reported in 12 studies, either as a numerical value (Vaquette et al., 2013; Wang et al., 2015; Kang et al., 2021) or described qualitatively as “adult” (Zhang P. et al., 2016) or “skeletally mature” (Li et al., 2017; Cai et al., 2018) or “mature” (Li et al., 2012; Jiang et al., 2014; Li et al., 2014; Ai et al., 2017; Cai et al., 2020; Wei et al., 2026).

Information on animal sex was provided in 12 studies, accounting for 432 animals (59.5% of the total), including 75 female animals (17.36%) (Vaquette et al., 2013; Li et al., 2017) and 357 male animals (82.64%) (Li et al., 2012; Jiang et al., 2014; Li et al., 2014; Wang et al., 2015; Ai et al., 2017; Cai et al., 2018; Yang et al., 2019; Cai et al., 2020; Kang et al., 2021; Wei et al., 2026). In all cases, animal sex was consistent within each study, with experimental groups comprising either exclusively female or exclusively male animal species.

Body weight was reported in 13 studies, either as mean values (Li et al., 2012; Jiang et al., 2014; Li et al., 2014; Wang et al., 2015; Ai et al., 2017; Wang S. et al., 2018; Yang et al., 2019; Cai et al., 2021; Kang et al., 2021) or ranges (Zhang P. et al., 2016; Cai et al., 2020; Zhang et al., 2020; Wei et al., 2026).

#### 3.3.5 Mechanical properties

An overview of the biomechanical properties evaluated across the included studies is presented in Table 4.

All included studies reported biomechanical testing, with substantial heterogeneity in the timing of assessment.

Only Li et al. (2012) performed biomechanical testing exclusively prior to implantation, assessing both tensile strength and strain, with no statistically significant differences observed between the experimental groups.

All the remaining studies conducted biomechanical evaluations after implantation, following graft harvesting from animal models (Vaquette et al., 2013; Jiang et al., 2014; Li et al., 2014; Wang et al., 2015; Zhang P. et al., 2016; Ai et al., 2017; Li et al., 2017; Cai et al., 2018; Wang S. et al., 2018; Yang et al., 2019; Cai et al., 2020; Zhang et al., 2020; Cai et al., 2021; Kang et al., 2021; Ma et al., 2021; Wei et al., 2026). The timing of post-implantation testing varied considerably across studies, ranging from minimum of 2 weeks (Li et al., 2014) to a maximum of 12 months (Vaquette et al., 2013).

Ultimate load to failure was the most frequently assessed biomechanical parameter and was reported in 16 studies (Vaquette et al., 2013; Jiang et al., 2014; Li et al., 2014; Wang et al., 2015; Zhang P. et al., 2016; Ai et al., 2017; Li et al., 2017; Cai et al., 2018; Wang S. et al., 2018; Yang et al., 2019; Cai et al., 2020; Zhang et al., 2020; Cai et al., 2021; Kang et al., 2021; Ma et al., 2021; Wei et al., 2026). Among these, two studies explicitly described pull-out as the failure mode, indicating that the reported ultimate load corresponded to pull-out strength (Li et al., 2014; Li et al., 2017).

Across all studies, the overall trend favored functionalized interventional grafts, which showed higher ultimate load to failure values, with differences ranging from modest, as in Wei et al. (2026) (40.8 ± 4.0 N vs. 37.1 ± 3.5 N at week 6), to substantial, as in Cai et al. (2018) (439.2 ± 27.3 N vs. 198.3 ± 17.8 N at week 12) (Cai et al., 2018) and Yang et al. (2019) (502.3 ± 20.0 N vs. 182.7 ± 14.5 N at week 16), corresponding to relative increases of up to approximately 2.7-fold over the control graft.

When compared with the native human ACL, lower ultimate load to failure values were reported for the functionalized graft (Wang et al., 2015; Cai et al., 2018), reaching approximately 70% of native ACL strength (Wang et al., 2015).

Stiffness was evaluated in 11 studies, with the overall trend favoring functionalized interventional grafts when compared to control grafts (Jiang et al., 2014; Wang et al., 2015; Zhang P. et al., 2016; Ai et al., 2017; Cai et al., 2018; Wang S. et al., 2018; Yang et al., 2019; Cai et al., 2020; Zhang et al., 2020; Cai et al., 2021; Wei et al., 2026).

When compared with human native ACL, lower stiffness values were reported for the functionalized graft (Wang et al., 2015; Cai et al., 2018), reaching approximately 46% of native ACL stiffness (Wang et al., 2015).

#### 3.3.6 *In vivo* outcomes

An overview of the *in vivo* outcomes evaluated across the included studies is presented in Table 5.

**TABLE 5:** *In vivo* outcomes.

Author, year	Animal model	Angiogenesis	Bone volume	New bone formation	Ligamentization	Interface width
(Li et al., 2012)	1) 30 New Zealand rabbits mature male, mean weight 3.2 ± 0.9 kg n = 15 rabbits per group Interventional group: HA-CG LBL-coated PET artificial ligament (HA-CG/pet) Control group: Uncoated PET artificial ligament (PET) 2) 6 GuangXi pigs mature male, mean weight 43.4 ± 8.5 kg n = 3 GuangXi pigs per group Interventional group: HA-CG LBL-coated PET artificial ligament (HA-CG/PET) Control group: Uncoated PET artificial ligament (PET)	At week 6 (qualitative histological observation): ↑ some vessels covered graft in HA-CG/PET vs. PET	NA	At week 12 (qualitative histological observation): ↑ collagen tissue formation in HA-CG/PET vs. PET	At week 12 (MRI misuration: mean signal/noise quotient SNQ): ↓ SNQ in HA-CG/PET (5.96 ± 0.81) vs. PET (9.28 ± 3.28)	NA
(Vaquette et al., 2013)	51 Pré-Alpes sheep female, 2 years old n = 28 Grafted Ligament n = 23 Non-Grafted Ligament; Interventional group: grafted LARS™ PET (GL) Control group: non-grafted LARS™ PET (NGL)	NA	At 3 and 12 months (qualitative histological observation) ↑ GL vs. NGL ligament to bone contact area	At 3 and 12 months (qualitative histological observation) no differences among GL vs. NGL	NA	NA
(Jiang et al., 2014)	72 New Zealand rabbits mature male, mean weight 2.8 ± 0.5 kg n = 24 per group, randomly divided Interventional group: SF/PET and SF-HAP/PET Control group: PET	NA	NA	At 3 and 12 months (qualitative histological observation) no differences among SF-HAP/PET vs. SF/PET and PET	NA	At week 4: ↓ SF-HA/PET (277.81 ± 41.34 µm) vs. SF/PET (308.19 ± 43.86 µm) and PET (315.00 ± 51.75 µm) At week 8 ↓↓ SF-HA/PET (139.19 ± 38.96 µm) vs. SF/PET (207.12 ± 44.49 µm) and PET (219.38 ± 47.59 µm)
(Li et al., 2014)	30 New Zealand rabbits mature male, mean weight 2.7 ± 0.6 kg n = 60 knee total, 30 for each group Interventional group: AKT/PET (right knee) Control group: PET (left knee)	NA	At week 4 (MICRO-CT): ↑↑ at graft-bone interface on AKT-PET (11.70 ± 2.35 mm^3^) vs. PET (9.10 ± 1.27 mm^3^) At week 8 (MICRO-CT): ↑↑ at graft-bone interface on AKT-PET (15.72 ± 2.83 mm^3^) vs. PET (10.66 ± 2.00 mm^3^)	At week 4 and 8 (qualitative histological observation): ↑ bone tissue formation in AKT-PET (+47%) vs. PET	NA	At week 4: ↓ AKT-PET (101,21 µm) vs. PET (155,81 µm) At week 8 ↓↓ AKT-PET (54,6 µm) vs. PET (136,50 µm)
(Wang et al., 2015)	32 New Zealand rabbits mature male, 12 weeks old, average weight 3.0 ± 0.4 kg n = 64 knees total, 32 per group Interventional group: G/PET Control group: PET (contralateral limb)	NA	At week 4 (MICRO-CT): ↑↑ BV/TV (%)) of G/PET (8.84 ± 0.34) vs. PET (3.43 ± 0.25) At week 8 (MICRO-CT) ↑↑ BV/TV (%) ofG/PET (19.16 ± 1.36) vs. PET (9.93 ± 0.75) At week 12 (MICRO-CT) ↑↑ BV/TV (%) of G/PET (26.43 ± 3.76) vs. PET (18.61 ± 1.47) At weeks 4, 8 and 12 ↑ TbTh and TbN; ↓ TbSp in G-PET-AL vs. PET-AL.	At week 8 (histological observation): ↑ BV/TV (%) of G/PET (18.74 ± 2.13) vs. PET (9.73 ± 1.24) ↑↑ MAR in G/PET (40.8 ± 3.5 μm/d) vs. PET (26.5 ± 2.8 μm/d) At week 12 (histological observation): ↑↑ BV/TV (%) of G/PET (29.82 ± 4.26) vs. PET (17.98 ± 2.17)	NA	NA
(Zhang et al., 2016b)	36 New Zealand White rabbits adult, average weight of 2.5–3.5 kg n = 12 rabbit for each group Interventional group: SIM-COL/PET, COL/PET Control group: PET	At week 4 and week 8 (histological observation) ↑ VEGF-positive staining in SIM-COL-PET vs. COL-PET vs. PET.	NA	NA	NA	At week 8 (qualitative histological observation) ↓ in SIM-COL-PET group vs. COL/PET and PET
(Ai et al., 2017)	46 New Zealand rabbits mature male, average weight 2.8 ± 0.5 Kg n = 23 per group, randomly divided Interventional group: SF-EDC/PET Control group: PET	NA	At week 12 (MICRO-CT average cross sectional area of bone tunnel): ↓ SF-EDC/PET (3.254 ± 0.128 cm2) vs. PET (4.250 ± 0.507 cm2)	NA	NA	At week 6: ↓↓ SF-EDC/PET (291.00 ± 6.24 µm) vs. PET (313.20 ± 5.45 µm) At week 12 ↓↓ SF-EDC/PET (175.40 ± 14.94 µm) vs. PET (242.20 ± 31.61 µm)
(Li et al., 2017)	24 goats healthy skeletally mature female n = 8 goats for each group Interventional groups: BG/PET and Cu-BG/PET Control group: PET	At 12 weeks (measured at graft-bone interface, technique: CD 31 immunofluorescence): ↑↑ Cu-BG/PET vs. BG/PET and PET	At 12 weeks (measured by MICRO-CT in the middle site (MS) of bone tunnel): ↑↑ Cu-BG/PET (127,10 mm^3^) vs. BG-PET (85,98 mm^3^) and PET (53,74 mm^3^) (	At 12 weeks MICRO-CT(measured in the middle site (MS) of bone tunnel) ↑↑ new bone width in Cu-BG/PET (52 ± 9 µm) vs. BG-PET (23 ± 8 µm) and PET (4 ± 4 µm) Qualitative histological analysis (at graft bone interface) ↑ new bone formation of Cu-BG/PET vs. BG-PET and PET	NA	At 12 weeks: ↓↓ Cu-BG/PET (60 ± 19 µm) vs. BG-PET (111 ± 31 µm) vs. PET (188 ± 53 µm)
(Cai et al., 2018)	24 beagle dogs male, healthy skeletally mature n = 24 dogs for each group n = 48 knee total, 24 for each group Interventional group: SF-HAP/PET Control group: PET	NA	At week 12 (MICRO-CT)) ↑↑ BV/TV (%) of SF-HAP/PET (23.3 ± 2.1) vs. PET (14.4 ± 2.5) ↓↓ bone tunnel area of SF-HAP/PET (20.5 ± 5.9 mm2) vs. PET (32.9 ± 10.1 mm2)	At week 12 (qualitative histological observation) ↑ SF-HAP/PET vs. PET	At week 12 (macroscopic observation): graft was well encapsulated by tendon-like tissue ↑ SF-HAP/PET vs. PET	(H) At week 6 ↓↓ in SF-HAP/PET (257.1 ± 35.9 µm) vs. PET (418.7 ± 50.9 µm) At week 12: ↓↓ in SF-HAP/PET (83.4 ± 21.1 µm) vs. PET (368.5 ± 34.8 µm)
(Wang et al., 2018)	72 New Zealand rabbits average weight of 2.9 ± 0.2 kg n = 24 per group, randomly divided Interventional group: HAP/PET-plasma and HAP/PET-immersed Control group: PET	NA	At week 12 (MICRO-CT Femoral bone tunnel area): ↓↓HAP/PET-plasma (4.79 ± 0.53 mm2) vs. PET (5.96 ± 0.58 mm2) ↓HAP/PET-plasma vs. HAP/PET-immersed At week 8 (MICRO-CT Tibial bone tunnel area): ↓↓HAP/PET-plasma (4.79 ± 0.43 mm^2^) vs. PET (5.81 ± 0.20 mm^2^), p<,0.05 ↓HAP/PET-plasma vs. HAP/PET immersed At week 12 (MICRO-CT tibial bone tunnel area): ↓↓HAP/PET-plasma (4.91 ± 0.33 mm^2^) vs. HAP/PET-immersed and PET (6.08 ± 0.19 mm^2^), p<,0.05	At week 12 (modified graft-to-bone histological score): ↑↑ HAP/PET-plasma vs. PET ↑ HAP/PET-immersed vs. PET	NA	At week 8 (histological score) ↑↑ HAP/PET-plasma (2,48) vs. PET (1.48) At week 12: ↑↑ HAP/PET-plasma (3.12) vs. PET (2,24)
(Yang et al., 2019)	24 beagle dogs male, healthy, average weight 12.40 ± 1.58 kg n = 12 dogs for each group Interventional group: HPC/PET Control group: PET	At week 4 and 16: (histologica evaluation): ↑mRNA ligamentization markers in HPC/PET vs. PET (At week 4: ↑↑ VEGF in HPC/PET (0.005 ± 0.002) vs. PET (0.003 ± 0.001); t week 16: ↑ VEGF expression in HPC/PET (0.006 ± 0.001) vs. PET (0.004 ± 0.001))	NA	At week 4:(Immunohistochemical staining): ↑↑ COL1 expression in HPC/PET (693.67 ± 45.98) vs. PET (516.67 ± 37.61) At week 16: ↑↑ COL1 expression in HPC/PET (732.33 ± 49.62) vs. PET (550.00 ± 43.49) ↑ expression of BMP-7, OCN e RUNX2 gene related	NA	NA
(Cai et al., 2020)	Forty-eight mature male New Zealand white rabbits, weight, 2.5–3.4 kg n = 24 for each gropu Interventional group: HAp–PET Control group: PET	NA	At week 12 (MICTO-CT) ↑↑ BV/TV (%) of HAp–PET (11.9 ± 2.2) vs. PET (8.8 ± 1.3) ↓↓ bone tunnel area of HAp–PET (7.3 ± 0.6 mm^2^) vs. PET (8.1 ± 0.3 mm^2^)	​	NA	At week 12 (qualitative histological observation) ↓ in HAP–PET vs. PET
(Zhang et al., 2020)	72 New Zealand white rabbits average weight of 2.5–3.5 kg n = 24 rabbits for each group Interventional group: PCL/PET, BMP-7/PCL/PET Control group: PET	At week 12 (gross observation, vessels): ↑ BMP-7/PCL/PET vs. PCL/PET vs. PET	At week 6 (MICRO-CT) ↑↑ BV/TV (%) of BMP-7/PCL/PET (17,46) vs. PCL/PET (8,08) vs. PET (4,09) At week 12 (MICRO-CT) ↑↑ BV/TV (%) of BMP-7/PCL/PET (28,53) vs. PCL/PET (10,97) vs. PET (11,87). At week 6 (MICRO-CT bone tunnel area): ↓↓ BMP-7/PCL/PET (2,19) vs. PCL/PET (2,64) vs. PET (3,07) At week 12 ↓ BMP-7/PCL/PET (1,36) vs. PCL/PET (1,74) vs. PET (2,64)	At week 12 (gross observation): ↑ BMP-7/PCL/PET vs. PCL/PET vs. PET	NA	At week 12: (qualitative histological observation) much narrower in BMP-7/PCL/PET group vs. PCL/PET and PET
(Cai et al., 2021)	60 New Zealand rabbits mean weight of 3.0 ± 0.5 kg n = 20 rabbits per group Interventional group: BM-CaP/PET, ED-CaP/PET Control group: PET	NA	At week 12 (MICRO-CT) ↑↑ BV/TV (%) of PET/BM-CaP (14.3 ± 3.4) and PET/ED-CaP (18.2 ± 4.4) vs. PET (10.1 ± 1.6), p < 0.05, P < 0.01 respectively) At week 12 (MICRO-CT bone tunnel area): ↓↓ PET/BM-CaP (5.1 ± 0.9 mm^2^) and PET/ED-CaP (4.0 ± 0.7 mm^2^) vs. PET (6.9 ± 0.4 mm^2^)	At week 12 (modified graft-to-bone score system): ↑ PET/ED-CaP vs. PET/BM-CaP vs. PET	NA	At week 6 and 12 (qualitative histological evaluation) ↓PET/ED-CaP vs. PET/BM-CaP and PET
(Kang et al., 2021)	15 New Zealand rabbits male, 10 weeks old, average weight 2.7 ± 0.3 kg n = 5 rabbits per group Interventional group: 500B-PDA/PET, PDA/PET Control group: PET	NA	At week 12 (MICRO-CT): ↑↑ BV/TV (%) of 500B/PDA/PET (18,49) vs. PDA/PET (12,68) vs. PET (8,31) At week 12 (MICRO-CT): ↓↓ BS/TV (1/mm) of 500B/PDA/PET (12,66) vs. PDA/PET (16,45) vs. PET (19,19)	NA	NA	NA
(Ma et al., 2021)	54 New Zealand rabbits n = 18 per groups Interventional group: HAP/PET, 2SrHAP/PET Control group: PET	NA	At week 4 (MICRO-CT) ↑↑ BV/TV (%) of 2SrHAP-PET (21,99) vs. HAP/PET (15,42) vs. PET (2,72). At week 8: ↑ BV/TV (%) 2SrHAP-PET (27,44) vs. HAP/PET (21,65) ↑↑ 2SrHAP-PET vs. PET (5,21) At week 12: ↑ BV/TV (%) 2SrHAP-PET (32,2) vs. HAP/PET (24,15) ↑↑ 2SrHAP-PET vs. PET (2,5)	At week 4 (histological score) ↑ 2SrHAP-PET (1.33) vs. HAP/PET (1) vs. PET (0.33). At week 8: ↑↑ 2SrHAP-PET (2) vs. HAP/PET (1) vs. PET (0.67) At week 12: ↑2SrHAP-PET (2.33) vs. HAp/PET (1.67) vs. PET (1)	NA	At week 8 (qualitative hystological evaluation) ↓ 2SrHAP-PET vs. HAP/PET and PET At week 12: ↓↓ 2SrHAP-PET vs. HAP/PET and PET
(Wei et al., 2026)	30 New Zealand white rabbit male, mature, weight 2.8–3.5 kg N = 15 per group (*via* computer-generated random number table) Interventional group: BP-SF/PET Control group: PET	NA	At week 12 (MICRO-CT): ↑↑ BV/TV (%) of BP/SF-PET (10.8 ± 1.6) vs. PET (8.5 ± 1.0), P < 0.05 At week 12 (MICRO-CT)): ↓↓ bone tunnel area BP/SF-PET (7.1 ± 0.3 mm^2^) vs. PET (7.8 ± 0.6 mm^2^), P < 0.05	At week 12 (histological score): ↑↑ BP/SF-PET (6 ± 1) vs. PET (3 ± 1), P < 0.01	NA	NA

2SrHAP-PET, PET, coated with 2% strontium-substituted hydroxyapatite; 500B-PDA/PET, BMP-2, immobilized PDA-coated PET; ACL, anterior cruciate ligament; AKT, akermanite; AKT-PET, Akermanite-coated PET; BG, bioactive glass; BM-CaP/PET, Biomineralized Calcium Phosphate-coated PET; BMP-2, Bone Morphogenetic Protein-2; BMP-7, Bone Morphogenetic Protein-7; BMP-7/PCL/PET, BMP-7-loaded PCL/PET, hybrid scaffold; BP, black phosphorous; BP/SF-PET, Black phosphorous and silk fibroin-coated PET; BS/TV, bone surface/total volume; BV/TV, bone volume/total volume; CaP, calcium phosphate; CD31, Cluster of Differentiation 31; CG, cationized gelatin; COL, collagen; COL1, Collagen type I; Cu-BG, Copper-containing Bioactive Glass; EDC, 1-Ethyl-3-(3-dimethylaminopropyl) carbodiimide; ED-CaP/PET, Electrodeposited Calcium Phosphate-coated PET; G/PET, Graphene-coated PET; GL, grafted ligament; H, histological analysis; HA, hyaluronic acid; HA-CG/PET, Hyaluronic Acid-Cationized Gelatin-coated PET; HAP, hydroxyapatite; HAP/PET-immersed, immersion-coated hydroxyapatite PET; HAP/PET-plasma, plasma spray-coated hydroxyapatite PET; HPC, hydroxypropylcellulose; LARS™, ligament advanced reinforcement system; LbL, Layer-by-Layer; MAR, mineral apposition rate; MICRO-CT, Micro-Computed Tomography; MRI, magnetic resonance imaging; NHS, N-Hydroxysuccinimide; NGL, Non-Grafted Ligament; PCL, polycaprolactone; PDA, polydopamine; PET, polyethylene terephthalate; SF, silk fibroin; SF-EDC/PET, Silk fibroin-coated PET, stabilized with EDC/NHS, crosslinking; SF-HAP/PET, Silk Fibroin/Hydroxyapatite-coated PET; SIM, simvastatin; SIM-COL/PET, Simvastatin-collagen-coated PET; SNQ, Signal-to-Noise Quotient; SrHAP, Strontium-substituted Hydroxyapatite; VEGF, vascular endothelial growth factor; TEM, transmission electron microscopy; XPS, X-ray Photoelectron Spectroscopy; XRD, X-ray Diffraction; ↓ lower (non significant); ↓↓ significantly lower; ↑ higher (non significant); ↑↑ significantly higher.

All included articles showed marked heterogeneity in animal models, experimental design, graft functionalization strategies, evaluation methods and timing of assessment.

Assessments were performed at early, intermediate and late time points, ranging from 4 weeks (Jiang et al., 2014; Wang et al., 2015; Zhang P. et al., 2016) to 12 weeks (Cai et al., 2018; Wang S. et al., 2018; Yang et al., 2019; Zhang et al., 2020; Cai et al., 2021; Kang et al., 2021; Ma et al., 2021; Wei et al., 2026) and from 3 to 12 months (Vaquette et al., 2013).

Across studies, *in vivo* analyses mainly combined micro-computed tomography (micro-CT), histological evaluation and, in some cases, immunohistochemistry and MRI to assess graft–bone healing, bone formation and ligamentization.

##### 3.3.6.1 Angiogenesis

Angiogenesis was evaluated in five studies (Li et al., 2012; Zhang P. et al., 2016; Li et al., 2017; Wang S. et al., 2018; Ma et al., 2021). The overall trend favored functionalized grafts, which showed an enhanced angiogenic response compared with unmodified PET controls. Li et al. (2012) qualitatively observed increased vessel coverage of the graft in HA-CG/PET compared with PET at week 6. Similarly, Cai et al. (2020) reported increased vessel formation around the graft at week 12 on gross observation, with the angiogenic response being higher in BMP-7/PCL/PET compared with both PCL/PET and PET. Li et al. (2017) reported significantly increased microvessel density at the graft–bone interface in Cu-BG/PET compared with both BG/PET and PET at 12 weeks, based on CD31 immunofluorescence. VEGF expression was evaluated in two studies (Zhang P. et al., 2016; Yang et al., 2019). Zhang P. et al. (2016) observed increased VEGF-positive staining in SIM-COL/PET compared with COL/PET and PET at both 4 and 8 weeks, while Yang et al. (2019) reported significantly increased VEGF expression in HPC/PET compared with PET at week 4, which remained elevated at week 16, although to a lesser extent.

##### 3.3.6.2 Bone tunnel area and bone formation

Bone-related outcomes were evaluated across the included studies through a combination of micro-CT and histological analyses, with a specific focus on bone volume fraction (BV/TV) and bone tunnel area. Micro-CT analysis was performed in eleven studies (Li et al., 2014; Wang et al., 2015; Ai et al., 2017; Cai et al., 2018; Wang S. et al., 2018; Cai et al., 2020; Zhang et al., 2020; Cai et al., 2021; Kang et al., 2021; Ma et al., 2021; Wei et al., 2026) with assessments typically conducted from 4 to 12 weeks postoperatively.

The overall trend favored functionalized grafts, which showed increased bone volume at the graft–bone interface following functionalization (Li et al., 2014; Wang et al., 2015; Li et al., 2017; Cai et al., 2018; Cai et al., 2020; Zhang et al., 2020; Cai et al., 2021; Kang et al., 2021).

Li et al. (2014) observed a significantly greater bone volume at the graft–bone interface in AKT/PET compared with PET, increasing from 11.70 ± 2.35 vs. 9.10 ± 1.27 mm^3^ at week 4–15.72 ± 2.83 vs. 10.66 ± 2.00 mm^3^ at week 8. Similarly, Li et al. (2017) reported a significantly higher bone volume at the middle site of the bone tunnel in Cu-BG/PET (127.10 mm^3^) compared with BG/PET (85.98 mm^3^) and PET (53.74 mm^3^) at 12 weeks. Wang et al. (2015) reported significantly higher BV/TV values in G/PET than PET at weeks 4, 8 and 12, increasing from 8.84% ± 0.34% vs. 3.43% ± 0.25% at week 4%–19.16% ± 1.36% vs. 9.93% ± 0.75% at week 8% and 26.43% ± 3.76% vs. 18.61% ± 1.47% at week 12.

A particularly pronounced increase in bone volume was reported by Zhang et al. (2020), who observed significantly higher BV/TV values in BMP-7/PCL/PET compared with PCL/PET and PET, with values of 17.46% vs. 8.08% vs. 4.09% at week 6% and 28.53% vs. 10.97% vs. 11.87% at week 12, respectively. Cai et al. (2018) similarly reported significantly higher BV/TV in SF-HAP/PET than PET at 12 weeks (23.3% ± 2.1% vs. 14.4% ± 2.5%). Cai et al. (2020) further reported significantly higher BV/TV in HAP-PET than PET at week 12 (11.9% ± 2.2% vs. 8.8% ± 1.3%). In another study, Cai et al. (2021) reported significantly increased BV/TV in both PET/BM-CaP (14.3% ± 3.4%) and PET/ED-CaP (18.2% ± 4.4%) compared with PET (10.1% ± 1.6%), with p < 0.05 and p < 0.01, respectively. Kang et al. (2021) also observed significantly higher BV/TV in 500B/PDA/PET (18.49%) compared with PDA/PET (12.68%) and PET (8.31%) at 12 weeks.

Additional micro-CT parameters further supported improved bone remodeling following functionalization. Wang et al. (2015) reported increased trabecular thickness and trabecular number, together with reduced trabecular separation, in G/PET compared with PET at weeks 4, 8 and 12. Kang et al. (2021) similarly reported a significantly lower bone surface-to-bone volume ratio (BS/TV) in 500B/PDA/PET (12.66 1/mm) compared with PDA/PET (16.45 1/mm) and PET (19.19 1/mm) at 12 weeks, consistent with a more mature and compact trabecular structure.

Reduced bone tunnel dimensions were also frequently reported following functionalization (Cai et al., 2018; Wang S. et al., 2018; Cai et al., 2020; Zhang et al., 2020; Cai et al., 2021). Cai et al. (2018) observed a significantly smaller combined femoral and tibial bone tunnel area in SF-HAP/PET than PET at 12 weeks (20.5 ± 5.9 vs. 32.9 ± 10.1 mm^2^). Ai et al. (2017) similarly reported a smaller average bone tunnel cross-sectional area in SF-EDC/PET compared with PET at 12 weeks (3.254 ± 0.128 vs. 4.250 ± 0.507 cm^2^). Zhang et al. (2020) observed a progressive reduction in bone tunnel area with BMP-7/PCL/PET, from 2.19 vs. 2.64 vs. 3.07 mm^2^ at week 6 to 1.36 vs. 1.74 vs. 2.64 mm^2^ at week 12 for BMP-7/PCL/PET, PCL/PET and PET, respectively. Wang S. et al. (2018) reported significantly smaller femoral tunnel areas for HAP/PET-plasma compared with PET at 12 weeks (4.79 ± 0.53 vs. 5.96 ± 0.58 mm^2^) and smaller tibial tunnel areas at weeks 8 and 12 (4.79 ± 0.43 vs. 5.81 ± 0.20 mm^2^ and 4.91 ± 0.33 vs. 6.08 ± 0.19 mm^2^, respectively). Cai et al. (2020) also reported a significantly smaller bone tunnel area in HAp-PET than PET at 12 weeks (7.3 ± 0.6 vs. 8.1 ± 0.3 mm^2^). Cai et al. (2021) reported similar findings for PET/BM-CaP and PET/ED-CaP, with bone tunnel areas of 5.1 ± 0.9 and 4.0 ± 0.7 mm^2^, respectively, compared with 6.9 ± 0.4 mm^2^ for PET.

Histological analyses further supported the micro-CT findings, demonstrating increased new bone formation and improved graft–bone contact in several functionalized constructs. Li et al. (2014) reported a 47% increase in bone tissue formation in the tunnel area with AKT/PET compared with PET at weeks 4 and 8. Li et al. (2017) reported a greater new bone width at the graft–bone interface in Cu-BG/PET (52 ± 9 µm) compared with BG/PET (23 ± 8 µm) and PET (4 ± 4 µm) at 12 weeks. Wang et al. (2015) reported higher histological BV/TV in G/PET than PET at week 8 (18.74% ± 2.13% vs. 9.73% ± 1.24%) and at week 12 (29.82% ± 4.26% vs. 17.98% ± 2.17%), together with a higher mineral apposition rate at week 8 (40.8 ± 3.5 vs. 26.5 ± 2.8 µm/day). Histological scoring further supported enhanced bone formation, with higher scores reported for HAP/PET-plasma than PET at week 12 (3.12 vs. 2.24) (Wang S. et al., 2018), for 2SrHAP/PET than HAP/PET and PET at week 12 (2.33 vs. 1.67 vs. 1.00) (Ma et al., 2021), and for BP-SF/PET than PET at 12 weeks (6 ± 1 vs. 3 ± 1) (Wei et al., 2026).

Ligamentization was evaluated in two studies using different assessment methods. Cai et al. (2018) performed a macroscopic evaluation at 12 weeks, reporting that grafts were well encapsulated by tendon-like tissue, with more evident and organized tissue coverage in SF-HAP/PET compared with PET. By contrast, Li et al. (2012) assessed ligamentization through MRI by measuring the signal/noise quotient (SNQ), and observed significantly lower SNQ values in HA-CG/PET (5.96 ± 0.81) compared with PET (9.28 ± 3.28), at 12 weeks, indicating reduced graft signal intensity and therefore more advanced graft maturation and remodeling.

##### 3.3.6.3 Interface width

Interface width was evaluated in eleven studies (Jiang et al., 2014; Li et al., 2014; Zhang P. et al., 2016; Ai et al., 2017; Li et al., 2017; Cai et al., 2018; Wang S. et al., 2018; Cai et al., 2020; Zhang et al., 2020; Cai et al., 2021; Ma et al., 2021), through histological analysis with assessments mainly performed at 4–12 weeks post-surgery. The overall trend favored functionalized grafts, which demonstrated a reduced graft–bone interface width compared with PET controls in most studies, indicating tighter integration and reduced fibrous tissue interposition. Six studies (Jiang et al., 2014; Li et al., 2014; Ai et al., 2017; Li et al., 2017; Cai et al., 2018; Wang S. et al., 2018) performed interface width evaluation using similar histomorphometric analyses, providing quantitative measurements of the graft–bone interface. By contrast, the remaining five studies relied on qualitative histological observations, reporting non-standardized assessments without the use of validated or comparable measurement methods (Zhang P. et al., 2016; Cai et al., 2020; Zhang et al., 2020; Cai et al., 2021; Ma et al., 2021).

Jiang et al. (2014) reported significantly lower interface widths for SF-HAP/PET compared with both SF/PET and PET, decreasing from 277.81 ± 41.34 µm vs. 315.00 ± 51.75 μm at week 4–139.19 ± 38.96 µm vs. 219.38 ± 47.59 μm at week 8. Li et al. (2014) similarly reported a reduction from 155.81 to 54.6 µm in AKT/PET compared with PET at week 8. Ai et al. (2017) observed significantly lower interface widths with SF-EDC/PET than PET, decreasing from 291.00 ± 6.24 vs. 313.20 ± 5.45 μm at week 6–175.40 ± 14.94 vs. 242.20 ± 31.61 μm at week 12.

A particularly marked reduction was reported by Cai et al. (2018), where interface width decreased from 418.7 ± 50.9 µm in PET to 257.1 ± 35.9 µm in SF-HAP/PET at week 6, and from 368.5 ± 34.8 to 83.4 ± 21.1 μm at week 12. Li et al. (2017) also reported significantly lower interface width with Cu-BG/PET (60 ± 19 µm) compared with BG/PET (111 ± 31 µm) and PET (188 ± 53 µm) at 12 weeks.

In addition to these quantitative assessments, five studies reported qualitatively narrower graft–bone interfaces following functionalization. Zhang P. et al. (2016) observed a much narrower interface in the SIM-COL/PET group compared with both COL/PET and PET at week 8 based on qualitative histological evaluation. Similarly, Zhang et al. (2020) reported a much narrower interface in the BMP-7/PCL/PET group compared with PCL/PET and PET at week 12 on histological observation. Cai et al. (2020) also reported a qualitative reduction in the graft–bone interface in HAP–PET compared with PET at week 12. Cai et al. (2021) further reported a qualitatively reduced interface in PET/ED-CaP compared with both PET/BM-CaP and PET at weeks 6 and 12. Similarly, Ma et al. (2021) observed a narrower graft–bone interface in 2SrHAP-PET compared with HAP/PET and PET at week 8, with a more pronounced reduction at week 12. Although these qualitative observations support the overall trend toward improved graft–bone integration, they should be interpreted separately from the quantitative interface-width measurements because of the lack of standardized measurement methods.

## 4 Discussion

The field of synthetic ligament engineering has undergone a substantial evolution over the past decade, shifting from the development of passive load-bearing substitutes toward the design of bioactive scaffolds capable of modulating cellular and tissue responses. The limited long-term success of first-generation polyethylene terephthalate (PET) ligaments, including early generations of the LARS™ system, has been largely attributed to inadequate biological integration at the graft–host interface. These shortcomings are closely related to the intrinsic bioinertness of PET, which is characterized by high hydrophobicity, limited protein adsorption, and the absence of biochemical cues required for cell adhesion, proliferation, and differentiation (Smol et al., 2022; Tulloch et al., 2019; Di Benedetto et al., 2020). The findings of the present systematic review indicate that surface biofunctionalization strategies can effectively overcome these limitations by transforming PET from a structurally competent but biologically passive material into a bioactive interface capable of promoting angiogenesis, osteogenesis, and graft–bone integration.

### 4.1 Mechanisms underlying the biological benefits of PET biofunctionalization

The overall pattern of improvements observed across the included studies suggests that the beneficial effects of PET functionalization are mediated by a common biological mechanism, despite the considerable heterogeneity of coating strategies. These approaches - mineral coatings, bioactive glasses, extracellular matrix-derived molecules, growth factors, and polymeric surface modifications - all shared the same underlying goal: modifying the physicochemical properties of the PET surface to regulate cell–material interactions.

Surface functionalization increases PET surface roughness, hydrophilicity, and the availability of biologically active binding sites. These changes, in turn, facilitate the adsorption of extracellular matrix proteins and promote integrin-mediated cell adhesion, which represents the first critical step in the establishment of a favorable regenerative microenvironment (Figure 3).

**FIGURE 3:**
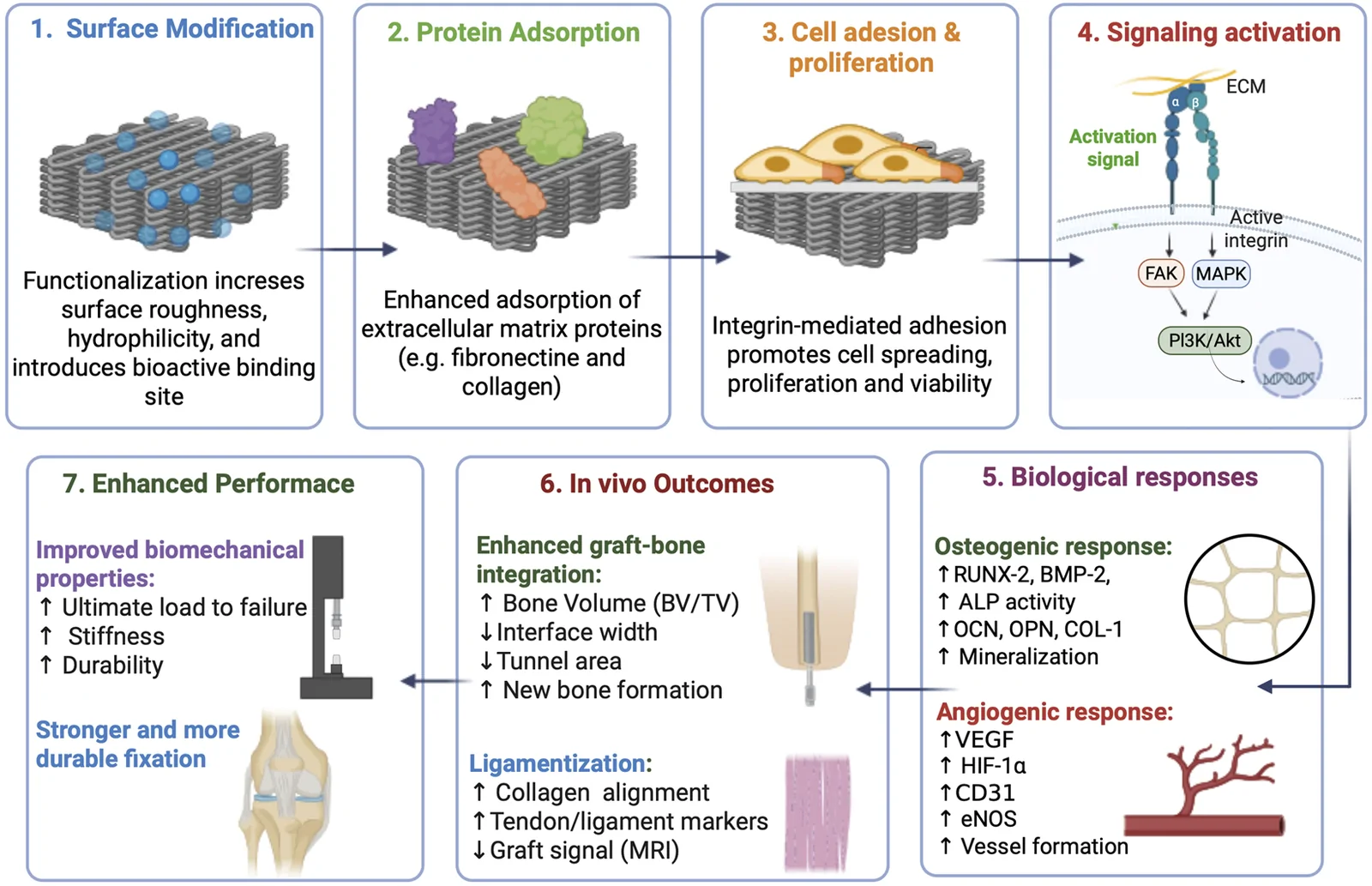
Proposed biological pathway underlying the enhanced performance of biofunctionalized PET scaffolds. Surface modification (1) increases roughness, hydrophilicity, and bioactive binding-site availability, promoting extracellular matrix protein adsorption (2) and integrin-mediated cell adhesion and proliferation (3). These early events are hypothesized to activate intracellular signaling pathways (4) driving osteogenic and angiogenic responses (5) These biological effects are associated with enhanced graft–bone integration *in vivo* and ligamentization, including collagen alignment, tendon/ligament marker expression, and reduced graft signal on MRI (6) which may in turn contribute to improved biomechanical properties and more durable fixation (7) This scheme represents a conceptual model derived from the cumulative evidence of the included studies rather than a demonstrated causal mechanism, as no single study directly tested the full proposed pathway.

Enhanced cell adhesion appears to trigger a cascade of downstream biological events: several studies included in this review reported increased cellular proliferation, viability, and spreading, which all are indicators of improved cytocompatibility in functionalized PET scaffolds. At the molecular level, these effects were associated with the upregulation of key osteogenic transcription factors and signaling molecules, including RUNX2 (Li et al., 2014; Wang et al., 2015; Zhang P. et al., 2016; Zhang et al., 2020; Kang et al., 2021), BMP-2 (Zhang P. et al., 2016; Ai et al., 2017; Li et al., 2017; Wang S. et al., 2018; Yang et al., 2019; Zhang et al., 2020; Ma et al., 2021), ALP (Vaquette et al., 2013; Jiang et al., 2014; Li et al., 2014; Zhang P. et al., 2016; Cai et al., 2018; Yang et al., 2019; Zhang et al., 2020; Cai et al., 2021; Kang et al., 2021; Ma et al., 2021), downstream signaling molecules such as p-ERK/ERK ratio (Cai et al., 2020), and late markers such as OCN, OPN, and COL1 (Li et al., 2014; Wang et al., 2015; Zhang P. et al., 2016; Ai et al., 2017; Li et al., 2017; Wang S. et al., 2018; Yang et al., 2019; Zhang et al., 2020; Cai et al., 2021; Kang et al., 2021; Ma et al., 2021; Wei et al., 2026), indicating activation of osteogenic differentiation pathways. This osteogenic response was frequently accompanied by increased mineralization and calcium deposition (Vaquette et al., 2013; Cai et al., 2018; Cai et al., 2020; Zhang et al., 2020; Kang et al., 2021; Ma et al., 2021; Wei et al., 2026), supporting the idea that biofunctionalization promotes the formation of a bone-like extracellular matrix at the graft–bone interface.

A similar trend emerges for angiogenesis, with *in vitro* upregulation of VEGF and other angiogenic markers reported in most studies (Wang et al., 2015; Zhang P. et al., 2016; Ai et al., 2017; Li et al., 2017; Cai et al., 2018; Wang S. et al., 2018; Cai et al., 2021; Kang et al., 2021; Ma et al., 2021), particularly at early time points (Li et al., 2017; Yang et al., 2019; Zhang et al., 2020). This is biologically meaningful, since vascular invasion is a prerequisite for bone formation and tissue remodeling: through the delivery of oxygen, nutrients, inflammatory mediators, and osteoprogenitor cells, newly formed vessels create the conditions necessary for effective graft incorporation. The concomitant upregulation of angiogenic and osteogenic markers suggests activation of the physiological angiogenesis–osteogenesis coupling process that characterizes normal bone healing (Grosso et al., 2017; Hu and Olsen, 2016).

These cellular and molecular changes in osteogenesis and angiogenesis were reflected in the *in vivo* findings. Functionalized grafts generally exhibited higher bone volume fraction (BV/TV) on micro-CT (Li et al., 2014; Wang et al., 2015; Li et al., 2017; Cai et al., 2018; Cai et al., 2020; Zhang et al., 2020; Cai et al., 2021; Kang et al., 2021) and reduced interface width at histological analysis, indicating closer graft–bone contact and enhanced bone formation at the interface (Jiang et al., 2014; Li et al., 2014; Zhang P. et al., 2016; Ai et al., 2017; Li et al., 2017; Cai et al., 2018; Wang S. et al., 2018; Cai et al., 2020; Zhang et al., 2020; Cai et al., 2021; Ma et al., 2021). Together, these findings are consistent with a more favorable biological environment for graft–bone integration following surface functionalization.

These biological improvements may contribute to the superior biomechanical performance observed in post-implantation testing. Notably, the absence of significant differences in pre-implantation mechanical properties, together with the overall improvement in mechanical outcomes observed after implantation, is consistent with the hypothesis that enhanced biological fixation may contribute to the biomechanical advantages of functionalized grafts. However, the available evidence does not allow this relationship to be considered causal, as none of the included studies directly investigated whether the degree of graft–bone integration mediated the observed changes in postoperative mechanical performance. Therefore, improved biological fixation should be regarded as a plausible contributing mechanism rather than a demonstrated primary determinant of mechanical performance.

Biomechanically, the overall trend across the included studies favored functionalized PET grafts over unmodified PET, particularly in terms of ultimate load to failure (Vaquette et al., 2013; Jiang et al., 2014; Li et al., 2014; Wang et al., 2015; Zhang P. et al., 2016; Ai et al., 2017; Li et al., 2017; Cai et al., 2018; Wang S. et al., 2018; Yang et al., 2019; Cai et al., 2020; Zhang et al., 2020; Cai et al., 2021; Kang et al., 2021; Ma et al., 2021; Wei et al., 2026) and stiffness (Jiang et al., 2014; Wang et al., 2015; Zhang P. et al., 2016; Ai et al., 2017; Cai et al., 2018; Wang S. et al., 2018; Yang et al., 2019; Cai et al., 2020; Zhang et al., 2020; Cai et al., 2021; Wei et al., 2026). Importantly, these improvements were observed predominantly in post-implantation testing, supporting a potential association between enhanced biological integration and postoperative mechanical performance rather than demonstrating that functionalization directly modifies the intrinsic mechanical properties of the scaffold.

This interpretation is supported by the fact that the only pre-implantation analysis did not show significant differences between groups (Li et al., 2012), while the large majority of post-implantation studies favored functionalized PET scaffolds.

Compared with the native ACL, however, functionalized PET grafts still demonstrated inferior mechanical properties, with lower ultimate load to failure and stiffness reported in direct comparisons (Wang et al., 2015; Cai et al., 2018), highlighting a persistent gap between synthetic constructs and native ligament tissue. Clinically, this suggests that, despite the gains achieved through functionalization, PET-based scaffolds are yet not fully able to replicate the mechanical behavior of the native ACL.

### 4.2 Design principles for next-generation synthetic ligaments

Despite the heterogeneity of the functionalization techniques employed, including physical deposition methods such as pulsed laser deposition of bioactive glass (Li et al., 2012; Li et al., 2014), chemical surface modifications such as plasma activation with layer-by-layer assembly (Li et al., 2012), and biomimetic coatings based on hydroxyapatite or calcium–phosphate deposition (Jiang et al., 2014; Cai et al., 2021; Ma et al., 2021), the overall trend across studies favored functionalized constructs, with most strategies aimed at increasing surface bioactivity resulting in improved cellular response and enhanced graft–bone integration. This suggests that the biological inertness of PET can be effectively addressed through surface functionalization, regardless of the specific technique used, since the response of host organisms to biomaterials is, in most cases, closely associated with the materials’ surface properties (Ma et al., 2007). Cell-surface interactions necessary for tissue engineering are limited on synthetic polymers by their smooth post-fabrication surfaces, hydrophobicity, and lack of recognizable biochemical binding sites (Richbourg et al., 2019); surface engineering of these bio-inert polymers is therefore needed to introduce bioactive species that promote cell adhesion, proliferation, viability, and enhanced extracellular matrix secretion (Ma et al., 2007; Richbourg et al., 2019). A rational combination of surface modification methods - including topographical modification, protein adsorption, mineral coating, functional group incorporation, and biomacromolecule immobilization - can provide nuanced, specific environments for targeted tissue development (Richbourg et al., 2019).

This also suggests that scaffold performance depends less on the base material itself than on its ability to provide bioactive cues at the interface. Evidence from studies on alternative materials, such as silk-based scaffolds, PCL, and carbon-based constructs, has similarly shown that the biofunctionalization significantly enhances osteointegration and biological response (Pearce et al., 2020; Reddy et al., 2021). This broader biomaterials literature is relevant to ACL reconstruction as it shows that different classes of scaffold materials can be engineered to overcome the limitations of their native physicochemical properties through appropriate functionalization.

Ebrahimi et al. (2022) created a scaffold based on PCL coated with collagen and hydroxyapatite (HAP), demonstrating that this functionalization significantly enhanced osteogenic differentiation of human adipose tissue-derived mesenchymal stem cells, with increased ALP activity, calcium deposition, and upregulation of bone-related genes compared to uncoated PCL. Similarly, Wang J. et al. (2018) developed a 3D-printed PCL scaffold functionalized with a porous collagen matrix, which promoted bone tissue regeneration and bone-material osteointegration compared to pristine PCL in critical-sized long bone defects. Font et al. (2018) fabricated biphasic silk fibroin scaffolds functionalized with heparin for growth factor delivery (TGF-β2 and GDF5), showing that heparin functionalization increased growth factor retention and synergistically enhanced tendon/ligament marker expression when combined with anisotropic pore alignment. Finally, Xue et al. (2022) combined silk fibroin with gelatin methacryloyl (GelMA) to create a co-electrospun nanofibrous scaffold, demonstrating enhanced mesenchymal stem cell growth, VEGF production, and tenogenic gene expression, with improved *in vivo* tendon tissue regeneration compared to silk fibroin alone.

Taken together, these studies provide an important comparison with the present PET-based approach. PCL, silk fibroin, and composite nanofibrous systems have been investigated not only for their capacity to support bone formation, but also for their ability to promote tendon/ligament-related cellular responses and tissue regeneration. The findings of Font et al. (2018) and Xue et al. (2022) are particularly relevant because they demonstrate that scaffold anisotropy, growth-factor retention, extracellular matrix-related functionalization, and tenogenic signaling can be exploited to reproduce aspects of the biological environment required for ligament regeneration. The present review focuses on graft–bone integration of PET-based ACL scaffolds, yet the broader ligament-engineering literature suggests that future ACL biomaterials may benefit from integrating osteogenic and angiogenic cues at the bone-facing region with tenogenic and mechanically oriented cues at the ligament-facing region.

These considerations also apply to the bone–ligament interface, a particularly challenging component of ACL reconstruction. Functionalization should therefore aim beyond promoting bone formation around the graft, toward establishing a biologically integrated transition between the ligament substitute and the surrounding bone. Combining mineral- or collagen-based osteogenic cues with anisotropic architecture and ligament-specific biological signals could provide a more biomimetic strategy for reproducing the graded environment of the native bone–ligament interface. While the studies included in the present review primarily assessed graft–bone integration rather than complete regeneration of a native enthesis, the reported improvements in osteogenesis, angiogenesis, and tendon/ligament marker expression support pursuing such multifunctional designs.

Comparison with the broader ligament-engineering and biomaterials literature suggests that next-generation ACL scaffolds should be designed as multifunctional systems rather than as uniformly bioactive materials. Spatially controlled functionalization may be particularly important, allowing the bone-facing portion of the scaffold to promote osteointegration while maintaining an appropriate biological and mechanical environment for ligament tissue formation along the intra-articular portion.

### 4.3 Limitations

Findings from this systematic review highlight several grey areas that currently limit the translation of current findings into clinical research. In particular, substantial heterogeneity was observed in both methodological approaches and data reporting across studies. From a methodological perspective, the included studies employed markedly different strategies to assess both *in vitro* and *in vivo* outcomes, thereby reducing comparability. For the same outcome domains, some investigations relied solely on qualitative descriptions, whereas others applied specific and validated quantitative assessment tools, further contributing to inconsistency in the reported findings.

Outcome reporting was also highly variable and often incomplete. Key parameters such as ligamentization and angiogenesis were assessed in only a small subset of *in vivo* studies, and in several cases the methodological details were insufficiently described, underscoring the need for more standardized and transparent reporting frameworks in this field. Interpretation of the results was further complicated by missing information regarding animal characteristics, including sample size, age, and body weight, as well as inadequate reporting of methodological rigor such as randomization, blinding procedures, and housing conditions, as also evidenced by the risk-of-bias assessment.

Importantly, the findings of this review should also be interpreted in light of the methodological limitations identified by the SYRCLE risk-of-bias assessment. Although most included studies reported beneficial effects of PET surface functionalization on bone formation, graft–bone integration, angiogenesis, or related outcomes, several domains were classified as having an unclear or high risk of bias. These limitations may affect the internal validity and reliability of the reported effects and therefore preclude definitive conclusions regarding the magnitude or consistency of the observed benefits. In addition, potential publication bias cannot be excluded, as studies reporting favorable effects of surface functionalization may be more likely to be published than studies showing null or negative findings. Given the limited number and heterogeneity of the included studies, a formal assessment of publication bias was not considered sufficiently reliable. Accordingly, the overall evidence should be regarded as promising but not conclusive, and the observed benefits of PET surface functionalization should be interpreted cautiously until confirmed by methodologically rigorous preclinical and clinical studies.

A further limitation is the substantial heterogeneity in biomarker assessment. ALP, RUNX2, and VEGF were evaluated using different methodologies and endpoints across studies, limiting direct comparisons and reducing the strength of quantitative conclusions. The adoption of standardized biomarker panels and reporting frameworks would improve the reproducibility and comparability of future investigations.

An additional limitation is the absence of a sex-based analytical approach. Most studies did not report the sex of the animals, while others demonstrated a clear sex bias by including only male or female subjects, limiting the generalizability of the findings. Furthermore, the use of different animal models - including goats, rabbits, and dogs - introduces additional heterogeneity that hampers direct comparison across studies.

A further critical gap is the limited use of large animal models that more closely replicate human knee joint anatomy, cartilage properties, and biomechanical loading conditions. Such limitations collectively reduce the ability to extrapolate preclinical findings to clinical settings.

Moreover, although several scaffold-based technologies showed promising results, the different scaffold types were tested under non-comparable conditions—different animal models, follow-up durations, and outcome measures—making it impossible to determine which approach was most effective.

For the same reason, meta-analysis was not performed: the substantial methodological and clinical heterogeneity - differences in intervention characteristics, animal models, outcome definitions, assessment methods, and follow-up time points - prevented a meaningful quantitative synthesis, and the findings of this review should be read in light of these constraints.

This lack of standardization and inconsistent reporting likely reflects the early developmental stage of this research field.

Nevertheless, these findings underscore the urgent need for standardized experimental models, harmonized outcome measures, and more rigorous reporting guidelines to improve the quality and translational value of future studies.

## 5 Conclusion

Functionalization of PET-based scaffolds generally enhances preclinical *in vitro* osteogenic and angiogenic activity, *in vivo* graft–bone integration, and post-implantation biomechanical performance compared with unmodified PET. These findings support the concept that surface biofunctionalization, rather than the base material itself, represents the key determinant of scaffold performance in ACL reconstruction.

However, the available evidence remains entirely preclinical and is predominantly derived from rabbit models. Moreover, only two studies directly compared functionalized PET scaffolds with the native ACL. Therefore, the current findings should be interpreted primarily as proof-of-concept evidence and cannot yet be directly extrapolated to clinical outcomes in humans. Nevertheless, these preclinical studies provide an essential foundation for formulating hypotheses and identifying the most promising strategies for subsequent clinical investigation.

Functionalized PET grafts still show inferior mechanical properties compared with the native ACL, highlighting a persistent gap between synthetic constructs and native ligament tissue. The heterogeneity of functionalization techniques, together with the absence of direct comparative studies, prevents the identification of a clearly superior strategy. Nevertheless, the evidence synthesized in this review suggests that the ideal synthetic ligament scaffold should combine high mechanical strength with a biologically active surface capable of promoting cell adhesion, angiogenesis, and osteogenesis, while ensuring long-term structural stability and minimizing debris generation.

### 5.1 Future development

Future research should focus on addressing the methodological and translational gaps identified in the present review. First, standardized experimental protocols and harmonized outcome measures should be established to improve comparability across studies, including consistent assessment of cellular responses, angiogenesis, osteogenesis, graft–bone integration, ligamentization, and biomechanical performance. Direct head-to-head comparisons of different functionalization strategies are also needed to identify which combinations of surface modification, bioactive molecules, and mineral or polymeric coatings provide the most favorable balance between biological activity and mechanical stability. In addition, the use of larger animal models and longer follow-up periods will be particularly important to evaluate graft maturation, long-term mechanical durability, inflammatory responses, wear debris generation, and overall safety under loading conditions more representative of the human knee. Finally, once sufficient preclinical evidence regarding safety, durability, and functional performance has been established, well-designed clinical studies will be required to determine whether the biological and biomechanical advantages observed in preclinical models translate into improved patient outcomes and support the clinical adoption of functionalized PET-based synthetic ligaments.

## Data Availability

The original contributions presented in the study are included in the article/supplementary material, further inquiries can be directed to the corresponding author.
